# Supplementary material for: Prevalence and incidence of neuromuscular conditions in the UK between 2000 and 2019: A retrospective study using primary care data
Source: PLoS One. 2021 Dec 31;16(12):e0261983. doi: 10.1371/journal.pone.0261983 (PMC8719665; doi:10.1371/journal.pone.0261983)
Supplement: S1 Table — (PDF) [file pone.0261983.s001.pdf]

Table S1 – Hierarchical structure of classifications based on available Read codes.

Level 1	Level 2	Level 3	Level 4
**Motor Neuron Disorders**	Spinal muscular atrophy		
	Motor neurone disease		
	Post-Polio syndrome		
**Muscle disease** (excluding Rhabdomyolysis)	Acquired myopathies	Endocrine myopathy	
		Infectious myopathy	
		Inflammatory myopathies	Dermatomyositis, Polymyositis
		Toxic or drug-induced myopathy	
	Hereditary myopathies	Congenital myopathies	Congenital muscular dystrophy, Congenital myopathy
		Metabolic myopathies	
		Muscular dystrophies	Becker, Duchenne, Emery–Dreifuss, Facioscapulohumeral, Limb girdle, Myotonic dystrophy Type 1, Oculopharyngeal
	Mitochondrial disease		
	Muscle channelopathies	Myotonic disorders (non-dystrophic)	Myotonia congenita
		Periodic paralysis	
	Myotonic disorders (unspecified)[1]		
**Neuropathy** (excluding Acquired & non-immune)	Hereditary Neuropathies	Charcot-Marie Tooth disease	
		Other	
	Inflammatory & autoimmune neuropathies	Guillain-Barré syndrome	
		Other	
**Neuromuscular Junction Disorder**	Lambert-Eaton syndrome		
	Myasthenia gravis		
	Other		
**Not specified**	Muscular or neuromuscular disease unspecified[2]		

GREY shading indicates the summary classification used in the analysis.

1 - This category only applies if they cannot be assigned to "Muscular Dystrophy" or "Myotonic Disorders (non-dystrophic)"

2 - This category only applies if they cannot be assigned to any of the above categories and mainly consists of "F39z.00 - Myopathy or muscular dystrophy NOS"
