# Supplementary material for: Prevalence and incidence of neuromuscular conditions in the UK between 2000 and 2019: A retrospective study using primary care data
Source: PLoS One. 2021 Dec 31;16(12):e0261983. doi: 10.1371/journal.pone.0261983 (PMC8719665; doi:10.1371/journal.pone.0261983)
Supplement: S2 Table — (PDF) [file pone.0261983.s002.pdf]

Table S2 – Read Codes used in classifying conditions.

Read Code	Term	Classification
AE1	Late effects of acute poliomyelitis	Post-Polio Syndrome
AyuJ5	[X]Sequelae of poliomyelitis	Post-Polio Syndrome
C3080	Medium chain acyl-CoA dehydrogenase deficiency	Metabolic myopathies
C3081	Multiple acyl-CoA dehydrogenase deficiencies	Metabolic myopathies
C310	Glycogenosis - glycogen storage disease	Metabolic myopathies
C310-1	Amylopectinosis	Metabolic myopathies
C310-3	Glycogen storage disease	Metabolic myopathies
C3100	McArdle's disease	Metabolic myopathies
C3100-1	Myophosphorylase deficiency	Metabolic myopathies
C3100-2	Glycogenosis, type 5	Metabolic myopathies
C3101	Generalised glycogenosis	Metabolic myopathies
C3101-2	Pompe's disease	Metabolic myopathies
C3101-3	Glycogenosis, type 2	Metabolic myopathies
C3103	Glycogenosis of liver and muscle	Metabolic myopathies
C3103-1	Glycogenosis of liver and muscle	Metabolic myopathies
C3103-3	Glycogenosis, type 3	Metabolic myopathies
C3104	Glycogenosis with hepatic cirrhosis	Metabolic myopathies
C3104-1	Glycogenosis, type 4	Metabolic myopathies
C3104-2	Andersen's disease	Metabolic myopathies
C310y	Other specified glycogenosis	Metabolic myopathies
C310z	Glycogenosis NOS	Metabolic myopathies
C3151	Mitochond encephalopathy, lact acidosis & strokelike episode	Mitochondrial disease
C3152	Kearns-Sayre syndrome	Mitochondrial disease
C3732	Familial neuropathic amyloid	Other Hereditary Neuropathy
C373E	Amyloid polyneuropathy type I	Other Hereditary Neuropathy
C373E-1	Swedish type amyloid polyneuropathy	Other Hereditary Neuropathy
C373E-2	Andrade type amyloid polyneuropathy	Other Hereditary Neuropathy
C373F	Familial amyloid polyneuropathy type II	Other Hereditary Neuropathy
C373F-1	Swiss type amyloid polyneuropathy	Other Hereditary Neuropathy
C373K	Familial amyloid polyneuropathy, Iowa type	Other Hereditary Neuropathy
C373K-1	Van Allen type amyloid polyneuropathy	Other Hereditary Neuropathy
C373K-2	British type amyloid polyneuropathy	Other Hereditary Neuropathy
C373K-3	Familial amyloid polyneuropathy type III	Other Hereditary Neuropathy
F10y0	Alper's disease	Mitochondrial disease
F10y1	Leigh's disease	Mitochondrial disease
F150	Werdnig - Hoffmann disease	Spinal Muscular Atrophy
F150-1	Infantile spinal muscular atrophy	Spinal Muscular Atrophy
F151	Spinal muscular atrophy	Spinal Muscular Atrophy
F1510	Unspecified spinal muscular atrophy	Spinal Muscular Atrophy
F1511	Kugelberg - Welander disease	Spinal Muscular Atrophy
F1511-1	Juvenile spinal muscular atrophy	Spinal Muscular Atrophy
F1512	Adult spinal muscular atrophy	Spinal Muscular Atrophy
F151z	Spinal muscular atrophy NOS	Spinal Muscular Atrophy
F152	Motor neurone disease	Motor Neurone Disease
F1520	Amyotrophic lateral sclerosis	Motor Neurone Disease
F1521	Progressive muscular atrophy	Motor Neurone Disease
F1521-1	Duchenne Aran muscular atrophy	Motor Neurone Disease
F1522	Progressive bulbar palsy	Motor Neurone Disease
F1523	Pseudobulbar palsy	Motor Neurone Disease
F1524	Primary lateral sclerosis	Motor Neurone Disease
F152z	Motor neurone disease NOS	Motor Neurone Disease
F29y1	Postpolio syndrome	Post-Polio Syndrome
F335	Neuralgic amyotrophy	Other Inflammatory & autoimmune neuropathy
F335-1	Parsonage - Aldren - Turner syndrome	Other Inflammatory & autoimmune neuropathy
F360	Hereditary peripheral neuropathy	Charcot-Marie Tooth

Read Code	Term	Classification
F3600	Dejerine-Sottas disease	Charcot-Marie Tooth
F360z	Hereditary peripheral neuropathy NOS	Charcot-Marie Tooth
F361	Peroneal muscular atrophy	Charcot-Marie Tooth
F3610	Charcot-Marie-Tooth disease	Charcot-Marie Tooth
F3610-2	Charcot-Marie-Tooth syndrome	Charcot-Marie Tooth
F361z	Peroneal muscular atrophy NOS	Charcot-Marie Tooth
F362	Hereditary sensory neuropathy	Charcot-Marie Tooth
F363	Refsum's disease	Other Hereditary Neuropathy
F365	Neuropathy in association with hereditary ataxia	Other Hereditary Neuropathy
F368	Hereditary motor and sensory neuropathy	Charcot-Marie Tooth
F3680	Hereditary motor and sensory neuropathy type I	Charcot-Marie Tooth
F3681	Hereditary motor and sensory neuropathy type II	Charcot-Marie Tooth
F3682	Hereditary motor and sensory neuropathy type III	Charcot-Marie Tooth
F3683	Hereditary motor and sensory neuropathy type IV	Other Hereditary Neuropathy
F370	Acute infective polyneuritis	Guillain-Barre
F3700	Guillain-Barre syndrome	Guillain-Barre
F3701	Postinfectious polyneuritis	Guillain-Barre
F3702	Miller-Fisher syndrome	Guillain-Barre
F370z	Acute infective polyneuritis NOS	Guillain-Barre
F371	Polyneuropathy in collagen vascular disease	Other Inflammatory & autoimmune neuropathy
F3710	Polyneuropathy in disseminated lupus erythematosus	Other Inflammatory & autoimmune neuropathy
F3712	Polyneuropathy in rheumatoid arthritis	Other Inflammatory & autoimmune neuropathy
F371z	Polyneuropathy in collagen vascular disease NOS	Other Inflammatory & autoimmune neuropathy
F3748	Polyneuropathy in porphyria	Other Inflammatory & autoimmune neuropathy
F3749	Polyneuropathy in sarcoidosis	Other Inflammatory & autoimmune neuropathy
F37X	Inflammatory polyneuropathy, unspecified	Other Inflammatory & autoimmune neuropathy
F38	Myoneural disorders	Other Neuromuscular Junction Disorder
F38-1	Neuromuscular disease	Muscular or Neuromuscular Disease unspecified
F380	Myasthenia gravis	Myasthenia Gravis
F3800	Persistent neonatal myasthenia gravis	Myasthenia Gravis
F3801	Juvenile or adult myasthenia gravis	Myasthenia Gravis
F380z	Myasthenia gravis NOS	Myasthenia Gravis
F381	Myasthenic syndrome due to disease EC	Other Neuromuscular Junction Disorder
F3810	Eaton-Lambert syndrome	Eaton-Lambert
F3810-1	Lambert-Eaton syndrome	Eaton-Lambert
F3811	Myasthenic syndrome due to other malignancy	Other Neuromuscular Junction Disorder
F3812	Myasthenic syndrome due to botulism	Other Neuromuscular Junction Disorder
F3814	Myasthenic syndrome due to hypothyroidism	Other Neuromuscular Junction Disorder
F3815	Myasthenic syndrome due to pernicious anaemia	Other Neuromuscular Junction Disorder
F3816	Myasthenic syndrome due to thyrotoxicosis	Other Neuromuscular Junction Disorder
F381z	Myasthenic syndrome due to disease NOS	Other Neuromuscular Junction Disorder
F382	Toxic myoneural disorder	Other Neuromuscular Junction Disorder
F383	Congenital and developmental myasthenia	Other Neuromuscular Junction Disorder
F38y	Other specific myoneural disorder	Other Neuromuscular Junction Disorder
F38y-1	Amyotonia congenita	Other Neuromuscular Junction Disorder
F38y-2	Oppenheim's amyotonia	Other Neuromuscular Junction Disorder
F38y-4	Floppy infant syndrome	Other Neuromuscular Junction Disorder
F38y-99	Other myoneural disorders	Other Neuromuscular Junction Disorder
F38z	Myoneural disorder NOS	Other Neuromuscular Junction Disorder
F39	Muscular dystrophies and other myopathies	Muscular dystrophy
F390	Congenital hereditary muscular dystrophy	Congenital myopathies & muscular dystrophies
F3900	Benign congenital myopathy	Congenital myopathies & muscular dystrophies
F3901	Central core disease	Congenital myopathies & muscular dystrophies
F3902	Centronuclear myopathy	Congenital myopathies & muscular dystrophies
F3903	Myotubular myopathy	Congenital myopathies & muscular dystrophies
F3904	Nemaline body disease	Congenital myopathies & muscular dystrophies
F3905	Congenital myopathy	Congenital myopathies & muscular dystrophies

Read Code	Term	Classification
F390z	Congenital hereditary muscular dystrophy NOS	Congenital myopathies & muscular dystrophies
F391	Hereditary progressive muscular dystrophy	Muscular dystrophy
F3910	Duchenne muscular dystrophy	Muscular dystrophy
F3910-1	Pseudohypertrophic dystrophy	Muscular dystrophy
F3911	Erb's muscular dystrophy	Muscular dystrophy
F3912	Pelvic muscular dystrophy	Muscular dystrophy
F3913	Other limb-girdle muscular dystrophy	Muscular dystrophy
F3914	Facioscapulohumeral muscular dystrophy	Muscular dystrophy
F3914-1	Facioscapulohumeral atrophy	Muscular dystrophy
F3915	Distal (Gower's) muscular dystrophy	Muscular dystrophy
F3916	Ocular muscular dystrophy	Muscular dystrophy
F3917	Oculopharyngeal muscular dystrophy	Muscular dystrophy
F3918	Becker muscular dystrophy	Muscular dystrophy
F3919	Hauptmann-Thannhauser muscular dystrophy	Muscular dystrophy
F391A	Emery-Dreifuss muscular dystrophy	Muscular dystrophy
F391B	Cardiomyopathy in Duchenne muscular dystrophy	Muscular dystrophy
F391y	Other specified hereditary progressive muscular dystrophy	Muscular dystrophy
F391y-1	Distal dystrophy	Muscular dystrophy
F391z	Hereditary progressive muscular dystrophy NOS	Muscular dystrophy
F392	Myotonic disorders	Myotonic Disorders (unspecified)
F3920	Dystrophia myotonica (Steinert's disease)	Muscular dystrophy
F3920-1	Steinert's disease	Muscular dystrophy
F3921	Myotonia congenita (Thomsen's disease)	Myotonic Disorders (non-dystrophic)
F3921-1	Thomsen's disease	Myotonic Disorders (non-dystrophic)
F3922	Paramyotonia congenita (Eulenburg's disease)	Myotonic Disorders (non-dystrophic)
F3922-1	Eulenburg's disease	Myotonic Disorders (non-dystrophic)
F3923	Infantile myotonia	Myotonic Disorders (non-dystrophic)
F3925	Proximal myotonic myopathy	Muscular dystrophy
F392y	Other specified myotonic disorder	Myotonic Disorders (unspecified)
F392z	Myotonic disorder NOS	Myotonic Disorders (unspecified)
F393	Familial periodic paralysis	Periodic paralysis
F393-1	Familial hypokalaemic periodic paralysis	Periodic paralysis
F394	Toxic myopathy	Toxic or drug-induced myopathy
F3940	Drug-induced myopathy	Toxic or drug-induced myopathy
F3941	Alcoholic myopathy	Toxic or drug-induced myopathy
F395	Myopathy due to endocrine disease EC	Endocrine myopathy
F3950	Myopathy due to Addison's disease	Endocrine myopathy
F3951	Myopathy due to Cushing's syndrome	Endocrine myopathy
F3952	Myopathy due to hypopituitarism	Endocrine myopathy
F3953	Myopathy due to myxoedema	Endocrine myopathy
F3954	Myopathy due to thyrotoxicosis	Endocrine myopathy
F395z	Myopathy due to endocrine disease NOS	Endocrine myopathy
F396	Symptomatic inflammatory myopathy in disease EC	Inflammatory myopathies
F3960	Myopathy due to amyloid	Inflammatory myopathies
F3961	Myopathy due to disseminated lupus erythematosus	Inflammatory myopathies
F3962	Myopathy due to malignant disease	Inflammatory myopathies
F3963	Myopathy due to polyarteritis nodosa	Inflammatory myopathies
F3964	Myopathy due to rheumatoid arthritis	Inflammatory myopathies
F3965	Myopathy due to sarcoidosis	Inflammatory myopathies
F3966	Myopathy due to scleroderma	Inflammatory myopathies
F3967	Myopathy due to Sjogren's disease	Inflammatory myopathies
F396z	Symptomatic inflammatory myopathy in disease NOS	Inflammatory myopathies
F398	Myopathy in metabolic diseases	Metabolic myopathies
F39B	Muscular dystrophy	Muscular dystrophy
F39W	Inflammatory myopathy, not elsewhere classified	Inflammatory myopathies
F39X	Mitochondrial myopathy, not elsewhere classified	Mitochondrial disease

Read Code	Term	Classification
F39y	Other myopathies and muscular dystrophies	Muscular dystrophy
F39z	Myopathy or muscular dystrophy NOS	Muscular or Neuromuscular Disease unspecified
F39z-99	Myopathy NOS	Muscular or Neuromuscular Disease unspecified
F4Gy1	Extraocular muscle myopathy	Muscular or Neuromuscular Disease unspecified
F4J71	Progressive external ophthalmoplegia	Mitochondrial disease
Fyu11	[X]Other inherited spinal muscular atrophy	Spinal Muscular Atrophy
Fyu71	[X]Other inflammatory polyneuropathies	Other Inflammatory & autoimmune neuropathy
Fyu77	[X]Polyneuropathy/systemic connective tissue disorders CE	Other Inflammatory & autoimmune neuropathy
Fyu7B	[X]Inflammatory polyneuropathy, unspecified	Other Inflammatory & autoimmune neuropathy
Fyu8	[X]Diseases of myoneural junction and muscle	Other Neuromuscular Junction Disorder
Fyu80	[X]Other specified myoneural disorders	Other Neuromuscular Junction Disorder
Fyu81	[X]Other primary disorders of muscles	Muscular or Neuromuscular Disease unspecified
Fyu82	[X]Other specified myopathies	Muscular or Neuromuscular Disease unspecified
Fyu83	[X]Myasthenic syndromes in endocrine diseases CE	Other Neuromuscular Junction Disorder
Fyu84	[X]Other myasthenic syndromes in neoplastic disease CE	Other Neuromuscular Junction Disorder
Fyu85	[X]Myasthenic syndromes/other diseases classified elsewhere	Other Neuromuscular Junction Disorder
Fyu86	[X]Myopathy/infectious+parasitic diseases CE	Infectious myopathy
Fyu87	[X]Myopathy in endocrine diseases classified elsewhere	Endocrine myopathy
Fyu88	[X]Myopathy in metabolic diseases classified elsewhere	Metabolic myopathies
Fyu89	[X]Myopathy in other diseases classified elsewhere	Muscular or Neuromuscular Disease unspecified
Fyu8A	[X]Mitochondrial myopathy, not elsewhere classified	Mitochondrial disease
Fyu8B	[X]Inflammatory myopathy, not elsewhere classified	Inflammatory myopathies
G552-1	Becker's disease	Myotonic Disorders (non-dystrophic)
G5581	Cardiomyopathy in myotonic dystrophy	Muscular dystrophy
G5582	Dystrophic cardiomyopathy	Muscular or Neuromuscular Disease unspecified
H57y1	Lung disease with polymyositis	Inflammatory myopathies
N003	Dermatomyositis	Inflammatory myopathies
N003-1	Poikilodermatomyositis	Inflammatory myopathies
N0030	Juvenile dermatomyositis	Inflammatory myopathies
N0031	Dermatopolymyositis in neoplastic disease	Inflammatory myopathies
N003X	Dermatopolymyositis, unspecified	Inflammatory myopathies
N004	Polymyositis	Inflammatory myopathies
N230	Infective myositis	Infectious myopathy
N230-1	Purulent myositis	Infectious myopathy
N230-2	Suppurative myositis	Infectious myopathy
N2300	Infective myositis-neck	Infectious myopathy
N2301	Infective myositis-back	Infectious myopathy
N2302	Infective myositis-shoulder	Infectious myopathy
N2303	Infective myositis-arm	Infectious myopathy
N2304	Infective myositis-forearm	Infectious myopathy
N2305	Infective myositis-hand	Infectious myopathy
N2306	Infective myositis-pelvis	Infectious myopathy
N2307	Infective myositis-thigh	Infectious myopathy
N2308	Infective myositis-leg	Infectious myopathy
N2309	Infective myositis-foot	Infectious myopathy
N230A	Muscle abscess	Infectious myopathy
N230B	Muscle abscess-neck	Infectious myopathy
N230C	Muscle abscess-back	Infectious myopathy
N230D	Muscle abscess-shoulder	Infectious myopathy
N230E	Muscle abscess-arm	Infectious myopathy
N230F	Muscle abscess-forearm	Infectious myopathy
N230G	Muscle abscess-hand	Infectious myopathy
N230H	Muscle abscess-pelvis	Infectious myopathy

Read Code	Term	Classification
N230J	Muscle abscess-thigh	Infectious myopathy
N230K	Muscle abscess-leg	Infectious myopathy
N230L	Muscle abscess-foot	Infectious myopathy
N2332	Myositis in sarcoidosis	Inflammatory myopathies
N2334	Antisynthetase syndrome	Inflammatory myopathies
N23y0	Interstitial myositis	Inflammatory myopathies
Nyu44	[X]Other dermatomyositis	Inflammatory myopathies
Nyu48	[X]Dermato(poly)myositis in neoplastic disease CE	Inflammatory myopathies
Nyu4E	[X]Dermatopolymyositis, unspecified	Inflammatory myopathies
Nyu86	[X]Myositis in bacterial diseases classified elsewhere	Infectious myopathy
Nyu87	[X]Myositis in protozoal and parasitic infections CE	Infectious myopathy
Nyu88	[X]Myositis in other infectious diseases CE	Infectious myopathy
Nyu89	[X]Myositis in sarcoidosis classified elsewhere	Inflammatory myopathies

Note: Two EMIS LV codes (which have no Read code equivalent) were additionally used in the definitions

- HNG0055 (Duchenne muscular dystrophy) = Muscular dystrophy
- HNG0179 (Neuromuscular condition) = Muscular or neuromuscular disease unspecified
