# Supplementary material for: Prevalence and incidence of neuromuscular conditions in the UK between 2000 and 2019: A retrospective study using primary care data
Source: PLoS One. 2021 Dec 31;16(12):e0261983. doi: 10.1371/journal.pone.0261983 (PMC8719665; doi:10.1371/journal.pone.0261983)
Supplement: S3 Table — (PDF) [file pone.0261983.s003.pdf]

Table S3 – Recent prevalence and incident rates for specific muscular dystrophies

Classification	n	All (95% CI)	Females (95%CI)	Males (95%CI)
**Prevalence in 2019 (per 100,000 persons)**				
- Duchenne MD	382	3.0 (2.7-3.3)	_	6.1 (5.5-6.7)
- Becker MD	260	2.1 (1.8-0.0)	_	4.1 (3.6-4.6)
- Limb-girdle MD*	175	1.4 (1.2-1.6)	1.3 (1.0-1.5)	1.5 (1.2-1.8)
- Facioscapulohumeral MD	392	3.1 (2.8-3.4)	2.7 (2.3-3.1)	3.5 (3.0-4.0)
- Myotonic dystrophy type 1†	1,178	9.3 (8.8-9.9)	9.2 (8.4-9.9)	9.5 (8.7-10.2)
**Incidence in 2015-9 (per 100,000 persons/year)**				
- Duchenne MD	82	0.13 (0.11-0.16)	_	0.27 (0.21-0.33)
- Becker MD	70	0.11 (0.09-0.14)	_	0.23 (0.18-0.28)
- Limb-girdle MD*	44	0.07 (0.05-0.09)	0.07 (0.04-0.10)	0.08 (0.04-0.11)
- Facioscapulohumeral MD	91	0.15 (0.12-0.18)	0.14 (0.10-0.18)	0.16 (0.11-0.20)
- Myotonic dystrophy type 1†	266	0.44 (0.38-0.49)	0.44 (0.36-0.51)	0.44 (0.36-0.51)

* - Includes codes for Erb's muscular dystrophy and Pelvic muscular dystrophy

† - Includes codes for dystrophia myotonica and Steinert's disease

Note: Read codes for Duchenne or Becker MD were not counted for females
