# Supplementary material for: Prevalence and incidence of neuromuscular conditions in the UK between 2000 and 2019: A retrospective study using primary care data
Source: PLoS One. 2021 Dec 31;16(12):e0261983. doi: 10.1371/journal.pone.0261983 (PMC8719665; doi:10.1371/journal.pone.0261983)
Supplement: S4 Table — (PDF) [file pone.0261983.s004.pdf]

Table S4 – Prevalence rates for recorded neuromuscular disease in 2019 by age group

Age in 2019	Inflammatory myopathies	Muscular dystrophies	Charcot-Marie Tooth disease	Guillain-Barré syndrome*		Myasthenia gravis	Motor neurone disease	Other	Total
				Last 5 years	>5 years old				
00-04	0.36	5.79	1.99	1.27	0.00	0.54	1.09	16.66	27.70
05-09	1.31	13.98	7.97	2.48	0.39	1.31	2.48	27.84	57.76
10-14	6.18	23.29	14.87	2.89	2.50	1.84	1.84	37.37	90.78
15-19	7.09	30.23	18.51	2.75	6.51	2.89	1.45	36.59	106.01
20-24	8.74	29.17	19.59	5.92	8.31	6.76	2.54	36.07	117.52
25-29	11.45	23.26	23.75	6.39	11.57	9.16	2.77	33.87	122.46
30-34	11.62	25.81	22.68	6.26	17.43	9.94	2.68	38.32	135.17
35-39	15.75	29.05	25.14	8.27	19.22	15.42	2.79	37.54	153.63
40-44	19.63	29.94	27.15	9.94	23.88	17.70	6.06	46.90	181.20
45-49	20.84	33.44	30.46	9.85	31.84	25.19	8.02	51.88	211.51
50-54	30.43	32.96	37.46	11.97	37.13	28.78	12.41	64.49	256.29
55-59	35.57	38.48	39.64	11.62	42.20	41.04	17.32	63.24	289.80
60-64	45.19	38.36	40.73	19.11	48.26	54.54	22.60	81.04	350.37
65-69	51.61	43.41	43.09	24.60	60.94	65.60	30.39	90.84	411.77
70-74	59.75	36.83	50.74	20.79	65.47	97.88	41.41	108.52	482.54
75-79	64.87	30.85	53.75	22.23	71.67	112.95	41.05	105.92	504.63
80-84	59.26	32.99	47.65	21.08	73.31	143.88	40.32	111.50	530.61
85-89	61.45	22.57	52.33	21.13	73.94	145.96	41.77	100.34	520.45
90-	40.27	15.95	37.23	11.40	79.01	129.16	26.59	72.18	411.78

Note: Prevalence rates are per 100,000 persons. Patients were assigned to one group only based on the most recent recorded code. Guillain-Barre syndrome was sub-divided as to whether a code was present in the last 5 years or not.
