# Supplementary material for: Prevalence and incidence of neuromuscular conditions in the UK between 2000 and 2019: A retrospective study using primary care data
Source: PLoS One. 2021 Dec 31;16(12):e0261983. doi: 10.1371/journal.pone.0261983 (PMC8719665; doi:10.1371/journal.pone.0261983)
Supplement: S5 Table — (PDF) [file pone.0261983.s005.pdf]

Table S5 – Prevalence rates for recorded neuromuscular disease in 2019 by region

Region		Inflammatory myopathies	Muscular dystrophies	Charcot-Marie Tooth disease	Guillain-Barré syndrome	Myasthenia gravis	Motor neurone disease	All Neuromuscular Disease
England (North)	Number of cases	587	820	707	1,000	857	338	5,824
	Std. Rate* (95%CI)	23.3 (21.4-25.2)	32.7 (30.5-34.9)	28.2 (26.1-30.3)	39.8 (37.4-42.3)	34.0 (31.7-36.3)	13.4 (11.9-14.8)	231.8 (225.8-237.7)
	Rate Ratio† (95%CI)	0.93 (0.86-1.01)	1.11 (1.03-1.18)	0.96 (0.89-1.03)	0.99 (0.93-1.05)	1.01 (0.94-1.08)	1.06 (0.95-1.18)	1.04 (1.01-1.06)
England (Midlands)	Number of cases	646	754	845	1,137	870	361	6,173
	Std. Rate* (95%CI)	24.3 (22.4-26.1)	29.1 (27.0-31.2)	32.3 (30.1-34.4)	42.8 (40.3-45.3)	32.1 (29.9-34.2)	13.3 (12.0-14.7)	233.0 (227.2-238.8)
	Rate Ratio† (95%CI)	0.97 (0.90-1.05)	0.99 (0.92-1.06)	1.09 (1.02-1.17)	1.07 (1.01-1.13)	0.95 (0.89-1.02)	1.06 (0.95-1.17)	1.04 (1.02-1.07)
England (South)	Number of cases	1,391	1,402	1,537	2,104	1,720	595	11,358
	Std. Rate* (95%CI)	26.7 (25.3-28.1)	26.3 (24.9-27.7)	29.1 (27.6-30.5)	40.3 (38.6-42.0)	33.5 (31.9-35.0)	11.6 (10.7-12.6)	216.8 (212.9-220.8)
	Rate Ratio† (95%CI)	1.07 (1.01-1.13)	0.89 (0.84-0.94)	0.99 (0.94-1.03)	1.00 (0.96-1.05)	0.99 (0.95-1.04)	0.92 (0.85-1.00)	0.97 (0.95-0.99)
Northern Ireland	Number of cases	49	79	51	77	92	30	467
	Std. Rate* (95%CI)	21.0 (15.2-26.9)	33.3 (26.0-40.7)	21.7 (15.8-27.7)	33.1 (25.7-40.5)	40.1 (31.9-48.3)	13.0 (8.4-17.7)	200.1 (182.0-218.3)
	Rate Ratio† (95%CI)	0.84 (0.61-1.08)	1.13 (0.88-1.38)	0.74 (0.53-0.94)	0.82 (0.64-1.01)	1.19 (0.95-1.44)	1.04 (0.67-1.41)	0.90 (0.81-0.98)
Scotland	Number of cases	252	413	286	429	437	140	2,556
	Std. Rate* (95%CI)	22.1 (19.4-24.9)	36.9 (33.4-40.5)	25.4 (22.5-28.4)	37.8 (34.2-41.4)	38.6 (35.0-42.2)	12.3 (10.2-14.3)	226.5 (217.7-235.3)
	Rate Ratio† (95%CI)	0.89 (0.78-1.00)	1.25 (1.13-1.37)	0.86 (0.76-0.96)	0.94 (0.85-1.03)	1.15 (1.04-1.25)	0.98 (0.81-1.14)	1.01 (0.97-1.05)
Wales	Number of cases	227	255	298	317	274	122	1,852
	Std. Rate* (95%CI)	26.0 (22.6-29.4)	30.5 (26.8-34.3)	35.1 (31.1-39.1)	36.5 (32.4-40.5)	30.7 (27.0-34.3)	13.7 (11.2-16.1)	214.5 (204.7-224.3)
	Rate Ratio† (95%CI)	1.04 (0.91-1.18)	1.04 (0.91-1.16)	1.19 (1.05-1.32)	0.91 (0.81-1.01)	0.91 (0.80-1.02)	1.09 (0.89-1.28)	0.96 (0.92-1.00)

Note: Prevalence rates are per 100,000 persons. Denominators in each region were: England (North) = 2,499,630, England (Midlands) = 2,579,345, England (South) = 5,385,014, Northern Ireland = 237,362, Scotland = 1,100,208, Wales = 823,728.
* - All rates have been age standardised to CPRD population as of 1/1/2019. † - This is the ratio compared to the estimate of the overall UK rate.
