# Supplementary material for: Prevalence and incidence of neuromuscular conditions in the UK between 2000 and 2019: A retrospective study using primary care data
Source: PLoS One. 2021 Dec 31;16(12):e0261983. doi: 10.1371/journal.pone.0261983 (PMC8719665; doi:10.1371/journal.pone.0261983)
Supplement: S6 Table — (PDF) [file pone.0261983.s006.pdf]

Table S6 – Incidence rates for recorded neuromuscular disease in 2015-2019 by region

Region		Inflammatory myopathies	Muscular dystrophies	Charcot-Marie Tooth disease	Guillain-Barré syndrome	Myasthenia gravis	Motor neurone disease	All Neuromuscular Disease
England (North)	Number of cases	136	154	176	220	297	423	1,806
	Std. Rate* (95%CI)	1.1 (0.9-1.3)	1.3 (1.1-1.5)	1.5 (1.2-1.7)	1.8 (1.6-2.1)	2.4 (2.2-2.7)	3.5 (3.1-3.8)	14.9 (14.3-15.6)
	Rate Ratio† (95%CI)	0.87 (0.72-1.01)	1.11 (0.94-1.29)	1.01 (0.86-1.16)	1.06 (0.92-1.21)	0.99 (0.88-1.10)	1.01 (0.91-1.11)	1.06 (1.01-1.11)
England (Midlands)	Number of cases	179	123	204	231	309	461	1,852
	Std. Rate* (95%CI)	1.4 (1.2-1.6)	1.0 (0.8-1.2)	1.6 (1.4-1.8)	1.8 (1.6-2.1)	2.4 (2.1-2.6)	3.5 (3.2-3.8)	14.4 (13.7-15.0)
	Rate Ratio† (95%CI)	1.08 (0.92-1.23)	0.85 (0.70-1.00)	1.11 (0.96-1.26)	1.06 (0.93-1.20)	0.96 (0.85-1.06)	1.01 (0.92-1.11)	1.02 (0.97-1.07)
England (South)	Number of cases	364	290	364	428	586	841	3,489
	Std. Rate* (95%CI)	1.4 (1.3-1.6)	1.1 (1.0-1.2)	1.4 (1.3-1.6)	1.7 (1.5-1.8)	2.4 (2.2-2.6)	3.4 (3.2-3.7)	13.9 (13.4-14.3)
	Rate Ratio† (95%CI)	1.11 (1.00-1.22)	0.96 (0.85-1.07)	0.98 (0.88-1.09)	0.99 (0.89-1.08)	0.96 (0.88-1.03)	1.00 (0.93-1.06)	0.98 (0.95-1.02)
Northern Ireland	Number of cases	8	14	16	16	34	38	141
	Std. Rate* (95%CI)	0.7 (0.2-1.3)	1.3 (0.6-1.9)	1.5 (0.8-2.2)	1.5 (0.8-2.2)	3.2 (2.2-4.3)	3.6 (2.5-4.8)	13.2 (11.0-15.4)
	Rate Ratio† (95%CI)	0.57 (0.18-0.97)	1.10 (0.52-1.68)	1.02 (0.52-1.52)	0.87 (0.44-1.29)	1.32 (0.87-1.76)	1.06 (0.72-1.40)	0.94 (0.78-1.09)
Scotland	Number of cases	52	89	64	103	166	176	746
	Std. Rate* (95%CI)	0.9 (0.7-1.2)	1.7 (1.3-2.0)	1.2 (0.9-1.5)	1.9 (1.5-2.2)	3.0 (2.6-3.5)	3.2 (2.7-3.7)	13.8 (12.8-14.7)
	Rate Ratio† (95%CI)	0.73 (0.53-0.93)	1.45 (1.15-1.76)	0.82 (0.62-1.03)	1.10 (0.89-1.32)	1.23 (1.05-1.42)	0.93 (0.79-1.07)	0.98 (0.91-1.05)
Wales	Number of cases	51	35	55	42	109	152	529
	Std. Rate* (95%CI)	1.2 (0.9-1.6)	0.9 (0.6-1.2)	1.4 (1.0-1.7)	1.0 (0.7-1.3)	2.5 (2.1-3.0)	3.5 (2.9-4.0)	12.7 (11.6-13.8)
	Rate Ratio† (95%CI)	0.95 (0.69-1.21)	0.77 (0.51-1.02)	0.94 (0.69-1.19)	0.60 (0.42-0.78)	1.03 (0.84-1.23)	1.02 (0.86-1.18)	0.90 (0.83-0.98)

Note: Incidence rates are per 100,000 persons and estimated from 2015-9 data per 100,000 person years. Denominators in each region were: England (North) = 11,973,195, England (Midlands) = 12,510,123, England (South) = 25,957,613, Northern Ireland = 1,092,650, Scotland = 5,310,757, Wales = 3,946,045.
* - All rates have been age standardised to CPRD population denominator for 2015-19. † - This is the ratio compared to the estimate of the overall UK rate.
