# Supplementary material for: Prevalence and incidence of neuromuscular conditions in the UK between 2000 and 2019: A retrospective study using primary care data
Source: PLoS One. 2021 Dec 31;16(12):e0261983. doi: 10.1371/journal.pone.0261983 (PMC8719665; doi:10.1371/journal.pone.0261983)
Supplement: S7 Table — (PDF) [file pone.0261983.s007.pdf]

Table S7 – Prevalence rates for recorded neuromuscular disease in 2019 by Index of Multiple Deprivation (England only)

IMD		Inflammatory myopathies	Muscular dystrophies	Charcot-Marie Tooth disease	Guillain-Barré syndrome	Myasthenia gravis	Motor neurone disease	All Neuromuscular Disease
1 (Least)	Number of cases	514	519	558	870	691	244	4,404
	Std. Rate* (95%CI)	26.6 (24.3-28.9)	28.5 (26.0-30.9)	29.8 (27.3-32.3)	45.1 (42.1-48.1)	34.6 (32.0-37.2)	12.2 (10.7-13.7)	230.2 (223.4-237.0)
	Rate Ratio† (95%CI)	1.04 (0.95-1.12)	0.98 (0.89-1.06)	1.00 (0.91-1.08)	1.08 (1.01-1.15)	1.03 (0.95-1.10)	0.93 (0.81-1.04)	1.01 (0.98-1.04)
2	Number of cases	433	454	499	751	596	225	3,820
	Std. Rate* (95%CI)	25.4 (23.0-27.8)	28.0 (25.4-30.6)	30.0 (27.4-32.7)	44.1 (40.9-47.2)	34.1 (31.3-36.8)	12.9 (11.2-14.5)	226.3 (219.1-233.5)
	Rate Ratio† (95%CI)	0.99 (0.90-1.08)	0.96 (0.87-1.05)	1.00 (0.92-1.09)	1.06 (0.98-1.13)	1.01 (0.93-1.09)	0.98 (0.85-1.11)	0.99 (0.96-1.03)
3	Number of cases	429	415	418	652	505	222	3,481
	Std. Rate* (95%CI)	27.8 (25.1-30.4)	27.4 (24.7-30.0)	27.3 (24.7-29.9)	42.2 (38.9-45.4)	32.4 (29.6-35.3)	14.3 (12.4-16.2)	226.4 (218.8-233.9)
	Rate Ratio† (95%CI)	1.08 (0.98-1.18)	0.94 (0.85-1.03)	0.91 (0.82-1.00)	1.01 (0.93-1.09)	0.96 (0.88-1.05)	1.09 (0.94-1.23)	0.99 (0.96-1.03)
4	Number of cases	340	452	448	538	430	160	3,125
	Std. Rate* (95%CI)	25.0 (22.3-27.6)	31.4 (28.5-34.3)	31.9 (28.9-34.8)	39.4 (36.0-42.7)	32.5 (29.5-35.6)	12.1 (10.3-14.0)	226.8 (218.8-234.7)
	Rate Ratio† (95%CI)	0.97 (0.87-1.08)	1.08 (0.98-1.18)	1.07 (0.97-1.16)	0.94 (0.86-1.02)	0.97 (0.87-1.06)	0.92 (0.78-1.07)	1.00 (0.96-1.03)
5 (Most)	Number of cases	267	413	391	410	383	165	2,766
	Std. Rate* (95%CI)	22.6 (19.9-25.3)	31.1 (28.1-34.1)	31.0 (27.9-34.1)	34.6 (31.3-38.0)	34.7 (31.2-38.2)	14.9 (12.6-17.2)	228.0 (219.5-236.5)
	Rate Ratio† (95%CI)	0.88 (0.78-0.99)	1.07 (0.96-1.17)	1.04 (0.93-1.14)	0.83 (0.75-0.91)	1.03 (0.93-1.13)	1.13 (0.96-1.31)	1.00 (0.96-1.04)

Note: Prevalence rates are per 100,000 persons. Denominators in each region were: IMD1 = 1,792,177, IMD2 = 1,598,934, IMD3 = 1,509,094, IMD4 = 1,456,511, IMD5 = 1,373,489.

* - All rates have been age standardised to CPRD population as of 1/1/2019. † - This is the ratio compared to the estimate of the overall UK rate.
