# Supplementary material for: Prevalence and incidence of neuromuscular conditions in the UK between 2000 and 2019: A retrospective study using primary care data
Source: PLoS One. 2021 Dec 31;16(12):e0261983. doi: 10.1371/journal.pone.0261983 (PMC8719665; doi:10.1371/journal.pone.0261983)
Supplement: S8 Table — (PDF) [file pone.0261983.s008.pdf]

Table S8 – Incidence rates for recorded neuromuscular disease in 2015-2019 Index of Multiple Deprivation (England only)

IMD		Inflammatory myopathies	Muscular dystrophies	Charcot-Marie Tooth disease	Guillain-Barré syndrome	Myasthenia gravis	Motor neurone disease	All Neuromuscular Disease
1 (Least)	Number of cases	130	94	147	198	234	364	1,398
	Std. Rate* (95%CI)	1.4 (1.2-1.7)	1.1 (0.9-1.3)	1.6 (1.4-1.9)	2.2 (1.9-2.5)	2.4 (2.1-2.7)	3.7 (3.3-4.1)	15.0 (14.2-15.8)
	Rate Ratio† (95%CI)	1.05 (0.87-1.23)	1.01 (0.81-1.21)	1.09 (0.91-1.27)	1.23 (1.06-1.40)	1.03 (0.90-1.16)	1.04 (0.93-1.14)	1.05 (0.99-1.10)
2	Number of cases	101	86	122	151	189	300	1,134
	Std. Rate* (95%CI)	1.2 (1.0-1.5)	1.1 (0.9-1.4)	1.5 (1.3-1.8)	1.9 (1.6-2.2)	2.2 (1.9-2.6)	3.5 (3.1-3.9)	13.9 (13.1-14.7)
	Rate Ratio† (95%CI)	0.92 (0.74-1.10)	1.05 (0.83-1.27)	1.03 (0.85-1.22)	1.06 (0.89-1.23)	0.95 (0.82-1.09)	0.98 (0.87-1.09)	0.97 (0.91-1.03)
3	Number of cases	105	75	108	107	185	284	1,067
	Std. Rate* (95%CI)	1.4 (1.1-1.7)	1.0 (0.8-1.3)	1.5 (1.2-1.7)	1.4 (1.2-1.7)	2.4 (2.1-2.8)	3.7 (3.3-4.2)	14.3 (13.5-15.2)
	Rate Ratio† (95%CI)	1.05 (0.85-1.25)	0.96 (0.74-1.18)	0.99 (0.80-1.17)	0.81 (0.66-0.97)	1.04 (0.89-1.19)	1.05 (0.93-1.17)	1.00 (0.94-1.06)
4	Number of cases	94	64	104	110	141	221	911
	Std. Rate* (95%CI)	1.4 (1.1-1.7)	0.9 (0.7-1.1)	1.5 (1.2-1.8)	1.6 (1.3-1.9)	2.2 (1.8-2.5)	3.5 (3.0-4.0)	13.7 (12.8-14.6)
	Rate Ratio† (95%CI)	1.04 (0.83-1.25)	0.84 (0.63-1.04)	1.03 (0.83-1.22)	0.91 (0.74-1.08)	0.93 (0.77-1.08)	0.98 (0.85-1.11)	0.96 (0.90-1.02)
5 (Most)	Number of cases	76	86	77	99	135	170	851
	Std. Rate* (95%CI)	1.3 (1.0-1.5)	1.2 (1.0-1.5)	1.2 (0.9-1.5)	1.6 (1.3-1.9)	2.5 (2.1-2.9)	3.3 (2.8-3.8)	14.4 (13.4-15.4)
	Rate Ratio† (95%CI)	0.94 (0.73-1.15)	1.15 (0.90-1.39)	0.82 (0.64-1.00)	0.91 (0.73-1.09)	1.05 (0.87-1.23)	0.92 (0.78-1.05)	1.01 (0.94-1.08)

Note: Incidence rates are per 100,000 persons and estimated from 2015-9 data per 100,000 person years. Denominators in each region were: IMD1 = 8,681,723, IMD2 = 8,663,828, IMD3 = 7,694,621, IMD4 = 7,288,595, IMD5 = 7,072,987.

* - All rates have been age standardised to CPRD population denominator for 2015-19. † - This is the ratio compared to the estimate of the overall UK rate.
