# Supplementary material for: Prevalence and incidence of neuromuscular conditions in the UK between 2000 and 2019: A retrospective study using primary care data
Source: PLoS One. 2021 Dec 31;16(12):e0261983. doi: 10.1371/journal.pone.0261983 (PMC8719665; doi:10.1371/journal.pone.0261983)
Supplement: S9 Table — (PDF) [file pone.0261983.s009.pdf]

Table S9 – Age standardised prevalence rates for all neuromuscular disease 2000-19

Year	Females				Males			
	Prevalence Rate (95%CI)	Rate Ratio (95%CI)	Prevalence Rate including GBS in last 5 years only* (95%CI)	Rate Ratio (95%CI)	Prevalence Rate (95%CI)	Rate Ratio (95%CI)	Prevalence Rate including GBS in last 5 years only (95%CI)	Rate Ratio (95%CI)
2000	129.2 (125.6-132.7)	0.62 (0.60-0.64)	112.9 (109.6-116.2)	0.62 (0.60-0.64)	144.0 (140.2-147.8)	0.60 (0.58-0.62)	124.4 (120.9-128.0)	0.60 (0.58-0.62)
2001	133.5 (130.1-136.9)	0.64 (0.62-0.66)	116.7 (113.5-119.9)	0.64 (0.62-0.67)	150.2 (146.5-153.9)	0.63 (0.61-0.65)	129.9 (126.5-133.4)	0.63 (0.61-0.65)
2002	140.8 (137.4-144.1)	0.68 (0.66-0.70)	123.3 (120.1-126.4)	0.68 (0.66-0.70)	156.4 (152.8-160.0)	0.65 (0.64-0.67)	135.5 (132.1-138.8)	0.65 (0.63-0.67)
2003	145.6 (142.3-148.9)	0.70 (0.68-0.72)	127.1 (124.0-130.1)	0.70 (0.68-0.72)	163.2 (159.6-166.7)	0.68 (0.66-0.70)	141.4 (138.1-144.7)	0.68 (0.66-0.70)
2004	153.1 (149.9-156.4)	0.74 (0.72-0.76)	134.0 (131.0-137.0)	0.74 (0.72-0.76)	169.6 (166.1-173.1)	0.71 (0.69-0.73)	147.0 (143.8-150.2)	0.71 (0.69-0.73)
2005	160.6 (157.3-163.8)	0.77 (0.75-0.79)	140.6 (137.6-143.6)	0.78 (0.75-0.80)	179.1 (175.6-182.6)	0.75 (0.73-0.77)	155.8 (152.5-159.0)	0.75 (0.73-0.77)
2006	166.3 (163.0-169.6)	0.80 (0.78-0.82)	145.4 (142.4-148.5)	0.80 (0.78-0.82)	185.8 (182.3-189.3)	0.78 (0.76-0.80)	161.5 (158.2-164.7)	0.78 (0.76-0.80)
2007	170.8 (167.6-174.1)	0.82 (0.80-0.84)	149.4 (146.3-152.5)	0.82 (0.80-0.85)	190.0 (186.5-193.5)	0.80 (0.78-0.81)	165.0 (161.7-168.2)	0.79 (0.77-0.81)
2008	173.6 (170.3-176.9)	0.83 (0.81-0.85)	152.0 (148.9-155.1)	0.84 (0.82-0.86)	193.7 (190.2-197.2)	0.81 (0.79-0.83)	167.5 (164.2-170.7)	0.81 (0.79-0.83)
2009	179.3 (176.0-182.6)	0.86 (0.84-0.88)	157.0 (154.0-160.1)	0.87 (0.84-0.89)	199.1 (195.5-202.6)	0.83 (0.81-0.85)	172.2 (168.9-175.5)	0.83 (0.81-0.85)
2010	183.4 (180.1-186.7)	0.88 (0.86-0.90)	160.5 (157.3-163.6)	0.88 (0.86-0.91)	205.3 (201.7-208.8)	0.86 (0.84-0.88)	177.5 (174.2-180.9)	0.85 (0.83-0.88)
2011	186.0 (182.7-189.3)	0.89 (0.87-0.92)	162.9 (159.8-166.0)	0.90 (0.87-0.92)	209.3 (205.8-212.9)	0.88 (0.86-0.90)	180.8 (177.5-184.1)	0.87 (0.85-0.89)
2012	188.7 (185.4-192.1)	0.91 (0.88-0.93)	165.2 (162.0-168.3)	0.91 (0.89-0.93)	213.5 (209.9-217.1)	0.89 (0.87-0.91)	184.6 (181.2-187.9)	0.89 (0.87-0.91)
2013	191.3 (187.9-194.7)	0.92 (0.90-0.94)	167.3 (164.1-170.4)	0.92 (0.90-0.95)	218.3 (214.6-221.9)	0.91 (0.89-0.93)	188.8 (185.4-192.2)	0.91 (0.89-0.93)
2014	196.0 (192.6-199.5)	0.94 (0.92-0.96)	171.1 (167.9-174.3)	0.94 (0.92-0.97)	223.3 (219.6-227.0)	0.93 (0.91-0.96)	193.0 (189.6-196.5)	0.93 (0.91-0.95)
2015	198.8 (195.3-202.3)	0.95 (0.93-0.98)	173.4 (170.2-176.6)	0.96 (0.93-0.98)	226.6 (222.9-230.3)	0.95 (0.93-0.97)	195.9 (192.4-199.3)	0.94 (0.92-0.97)
2016	200.7 (197.2-204.2)	0.96 (0.94-0.99)	175.0 (171.7-178.2)	0.96 (0.94-0.99)	230.1 (226.4-233.9)	0.96 (0.94-0.99)	199.5 (196.0-203.0)	0.96 (0.94-0.98)
2017	201.6 (198.1-205.1)	0.97 (0.94-0.99)	176.0 (172.7-179.2)	0.97 (0.94-1.00)	234.8 (231.0-238.6)	0.98 (0.96-1.01)	203.6 (200.1-207.2)	0.98 (0.96-1.00)
2018	204.4 (200.9-207.9)	0.98 (0.96-1.01)	178.2 (174.9-181.5)	0.98 (0.96-1.01)	236.0 (232.2-239.8)	0.99 (0.97-1.01)	204.9 (201.4-208.4)	0.99 (0.96-1.01)
2019	208.3 (204.8-211.9)	1	181.4 (178.1-184.7)	1	239.0 (235.1-242.8)	1	207.8 (204.2-211.4)	1

Note: Prevalence rates are per 100,000 persons. All rates have been age standardised to CPRD population as of 1/1/2019.
* Only Guillain-Barré syndrome codes recorded in last 5 years are counted here.
