# Supplementary material for: Prevalence and incidence of neuromuscular conditions in the UK between 2000 and 2019: A retrospective study using primary care data
Source: PLoS One. 2021 Dec 31;16(12):e0261983. doi: 10.1371/journal.pone.0261983 (PMC8719665; doi:10.1371/journal.pone.0261983)
Supplement: S10 Table — (PDF) [file pone.0261983.s010.pdf]

Table S10 – Age standardised incidence rates for all neuromuscular disease 2000-19

Year	Females		Males	
	**Incidence Rate (95%CI)**	**Rate Ratio (95% CI)**	**Incidence Rate (95%CI)**	**Rate Ratio (95% CI)**
2000	11.5 (10.5-12.6)	0.93 (0.82-1.05)	15.0 (13.7-16.2)	1.01 (0.90-1.12)
2001	12.7 (11.7-13.8)	1.03 (0.92-1.15)	15.4 (14.3-16.6)	1.04 (0.94-1.15)
2002	12.1 (11.1-13.1)	0.98 (0.87-1.09)	16.0 (14.8-17.1)	1.07 (0.97-1.19)
2003	13.8 (12.8-14.8)	1.12 (1.00-1.24)	15.6 (14.5-16.7)	1.05 (0.95-1.16)
2004	13.4 (12.4-14.3)	1.08 (0.97-1.20)	16.0 (14.9-17.1)	1.08 (0.98-1.19)
2005	13.1 (12.2-14.1)	1.06 (0.95-1.18)	15.7 (14.7-16.7)	1.06 (0.96-1.16)
2006	12.7 (11.8-13.6)	1.03 (0.92-1.14)	14.9 (13.9-15.9)	1.00 (0.91-1.11)
2007	12.0 (11.1-12.9)	0.97 (0.87-1.08)	14.7 (13.7-15.7)	0.99 (0.90-1.09)
2008	13.0 (12.1-13.9)	1.05 (0.95-1.16)	14.9 (13.9-15.9)	1.00 (0.91-1.11)
2009	12.3 (11.4-13.2)	0.99 (0.89-1.10)	15.3 (14.3-16.2)	1.03 (0.93-1.13)
2010	12.4 (11.6-13.3)	1.01 (0.91-1.12)	15.2 (14.3-16.2)	1.03 (0.93-1.13)
2011	11.5 (10.7-12.4)	0.93 (0.84-1.04)	14.5 (13.6-15.5)	0.98 (0.89-1.08)
2012	12.6 (11.7-13.4)	1.01 (0.91-1.12)	15.9 (15.0-16.9)	1.07 (0.98-1.18)
2013	12.8 (11.9-13.7)	1.03 (0.93-1.14)	15.3 (14.3-16.3)	1.03 (0.94-1.13)
2014	12.0 (11.1-12.8)	0.97 (0.87-1.07)	14.9 (13.9-15.9)	1.00 (0.91-1.10)
2015	12.1 (11.2-13.0)	0.98 (0.88-1.09)	17.2 (16.1-18.2)	1.15 (1.05-1.27)
2016	12.1 (11.2-12.9)	0.97 (0.88-1.08)	16.5 (15.5-17.5)	1.11 (1.01-1.22)
2017	12.8 (11.9-13.7)	1.03 (0.93-1.14)	15.5 (14.5-16.5)	1.04 (0.95-1.15)
2018	13.1 (12.2-14.0)	1.06 (0.95-1.17)	16.2 (15.2-17.2)	1.09 (0.99-1.20)
2019	12.4 (11.4-13.3)	1	14.9 (13.8-15.9)	1

Note: Incidence rates are per 100,000 person years. All rates have been age standardised to CPRD population as of 1/1/2019.
