# Supplementary material for: Prevalence and incidence of neuromuscular conditions in the UK between 2000 and 2019: A retrospective study using primary care data
Source: PLoS One. 2021 Dec 31;16(12):e0261983. doi: 10.1371/journal.pone.0261983 (PMC8719665; doi:10.1371/journal.pone.0261983)
Supplement: S11 Table — (PDF) [file pone.0261983.s011.pdf]

Table S11 – Age standardised lifetime prevalence rates 2000-19 for all neuromuscular disease in females by age

Year	Females 0-14 years		Females 15-44 years		Females 45-64 years		Females 65+ years	
	Prevalence Rate (95%CI)	Rate Ratio (95%CI)	Prevalence Rate (95%CI)	Rate Ratio (95%CI)	Prevalence Rate (95%CI)	Rate Ratio (95%CI)	Prevalence Rate (95%CI)	Rate Ratio (95%CI)
2000	31.9 (27.2-36.6)	0.71 (0.60-0.85)	90.3 (85.7-94.9)	0.70 (0.66-0.75)	183.2 (174.7-191.7)	0.69 (0.65-0.73)	210.7 (200.8-220.7)	0.50 (0.48-0.53)
2001	31.6 (27.2-36.1)	0.71 (0.60-0.83)	92.7 (88.3-97.2)	0.72 (0.68-0.77)	188.3 (180.2-196.5)	0.71 (0.67-0.75)	221.4 (211.7-231.2)	0.53 (0.50-0.56)
2002	33.8 (29.5-38.2)	0.75 (0.64-0.88)	96.8 (92.5-101.2)	0.76 (0.71-0.80)	196.6 (188.7-204.6)	0.74 (0.70-0.78)	237.4 (227.7-247.1)	0.57 (0.54-0.60)
2003	36.1 (31.8-40.5)	0.81 (0.69-0.94)	99.9 (95.7-104.2)	0.78 (0.74-0.82)	201.8 (194.1-209.6)	0.76 (0.72-0.80)	247.0 (237.4-256.5)	0.59 (0.56-0.62)
2004	36.6 (32.4-40.8)	0.82 (0.71-0.95)	105.1 (100.9-109.3)	0.82 (0.78-0.87)	211.0 (203.4-218.6)	0.79 (0.76-0.83)	262.5 (252.9-272.0)	0.63 (0.60-0.66)
2005	38.7 (34.5-42.9)	0.86 (0.75-1.00)	109.0 (104.9-113.2)	0.85 (0.81-0.90)	221.1 (213.6-228.6)	0.83 (0.79-0.87)	277.5 (267.8-287.1)	0.66 (0.64-0.69)
2006	39.7 (35.5-43.9)	0.89 (0.77-1.02)	112.3 (108.1-116.5)	0.88 (0.83-0.92)	227.6 (220.1-235.1)	0.86 (0.82-0.89)	290.7 (281.0-300.5)	0.70 (0.67-0.73)
2007	39.9 (35.7-44.1)	0.89 (0.77-1.02)	114.3 (110.1-118.5)	0.89 (0.85-0.94)	230.0 (222.5-237.5)	0.86 (0.83-0.90)	306.4 (296.4-316.3)	0.73 (0.70-0.77)
2008	40.7 (36.6-44.9)	0.91 (0.79-1.04)	113.4 (109.2-117.5)	0.89 (0.84-0.93)	235.8 (228.3-243.3)	0.89 (0.85-0.93)	313.7 (303.7-323.8)	0.75 (0.72-0.78)
2009	42.3 (38.1-46.4)	0.94 (0.82-1.08)	115.3 (111.1-119.4)	0.90 (0.86-0.95)	241.5 (234.0-249.0)	0.91 (0.87-0.95)	329.9 (319.7-340.1)	0.79 (0.76-0.82)
2010	44.7 (40.5-49.0)	1.00 (0.87-1.14)	115.9 (111.7-120.1)	0.91 (0.86-0.95)	245.9 (238.4-253.4)	0.92 (0.89-0.96)	341.7 (331.4-352.0)	0.82 (0.79-0.85)
2011	44.3 (40.1-48.5)	0.99 (0.87-1.13)	116.2 (112.0-120.4)	0.91 (0.86-0.95)	248.1 (240.6-255.6)	0.93 (0.89-0.97)	351.6 (341.2-362.0)	0.84 (0.81-0.88)
2012	45.9 (41.6-50.1)	1.02 (0.90-1.17)	116.7 (112.5-120.9)	0.91 (0.87-0.96)	247.5 (239.9-255.0)	0.93 (0.89-0.97)	364.0 (353.5-374.5)	0.87 (0.84-0.91)
2013	47.0 (42.7-51.3)	1.05 (0.92-1.19)	116.8 (112.6-121.0)	0.91 (0.87-0.96)	249.5 (242.0-257.0)	0.94 (0.90-0.98)	373.1 (362.5-383.6)	0.89 (0.86-0.93)
2014	47.2 (42.9-51.5)	1.05 (0.93-1.20)	120.5 (116.2-124.9)	0.94 (0.89-0.99)	253.2 (245.5-260.9)	0.95 (0.91-0.99)	384.7 (373.9-395.5)	0.92 (0.89-0.96)
2015	47.4 (43.1-51.7)	1.06 (0.93-1.20)	124.0 (119.5-128.4)	0.97 (0.92-1.02)	255.9 (248.2-263.6)	0.96 (0.92-1.00)	388.4 (377.6-399.3)	0.93 (0.90-0.97)
2016	44.7 (40.6-48.8)	1.00 (0.88-1.14)	124.1 (119.6-128.6)	0.97 (0.92-1.02)	261.3 (253.5-269.1)	0.98 (0.94-1.02)	392.5 (381.5-403.5)	0.94 (0.90-0.98)
2017	45.1 (40.9-49.2)	1.01 (0.88-1.15)	123.7 (119.3-128.2)	0.97 (0.92-1.02)	260.8 (253.0-268.6)	0.98 (0.94-1.02)	398.2 (387.1-409.3)	0.95 (0.92-0.99)
2018	45.3 (41.1-49.4)	1.01 (0.89-1.15)	124.5 (120.0-129.0)	0.97 (0.92-1.02)	263.2 (255.3-271.0)	0.99 (0.95-1.03)	407.8 (396.6-418.9)	0.98 (0.94-1.02)
2019	44.8 (40.7-48.9)	1	128.1 (123.5-132.6)	1	265.9 (258.1-273.8)	1	417.3 (406.0-428.6)	1

Note: All rates are per 100,000 persons and have been age standardised to CPRD population as of 1/1/2019
