# Supplementary material for: Prevalence and incidence of neuromuscular conditions in the UK between 2000 and 2019: A retrospective study using primary care data
Source: PLoS One. 2021 Dec 31;16(12):e0261983. doi: 10.1371/journal.pone.0261983 (PMC8719665; doi:10.1371/journal.pone.0261983)
Supplement: S12 Table — (PDF) [file pone.0261983.s012.pdf]

Table S12 – Age standardised lifetime prevalence rates 2000-19 for all neuromuscular disease in males by age

Year	Males 0-14 years		Males 15-44 years		Males 45-64 years		Males 65+ years	
	Prevalence Rate (95%CI)	Rate Ratio (95%CI)	Prevalence Rate (95%CI)	Rate Ratio (95%CI)	Prevalence Rate (95%CI)	Rate Ratio (95%CI)	Prevalence Rate (95%CI)	Rate Ratio (95%CI)
2000	59.2 (53.3-65.2)	0.76 (0.67-0.86)	91.6 (87.0-96.2)	0.62 (0.59-0.66)	186.7 (178.3-195.2)	0.67 (0.63-0.70)	278.2 (265.0-291.3)	0.52 (0.49-0.54)
2001	61.3 (55.5-67.1)	0.78 (0.70-0.88)	97.6 (93.1-102.1)	0.67 (0.63-0.70)	190.8 (182.8-198.9)	0.68 (0.65-0.72)	292.1 (279.3-305.0)	0.54 (0.51-0.57)
2002	60.2 (54.8-65.7)	0.77 (0.69-0.86)	102.7 (98.3-107.1)	0.70 (0.66-0.74)	196.7 (188.9-204.4)	0.70 (0.67-0.74)	308.9 (296.3-321.5)	0.57 (0.54-0.60)
2003	60.6 (55.2-65.9)	0.77 (0.69-0.87)	106.2 (101.9-110.5)	0.72 (0.69-0.76)	204.3 (196.7-212.0)	0.73 (0.70-0.76)	327.9 (315.3-340.6)	0.61 (0.58-0.64)
2004	61.1 (56.0-66.2)	0.78 (0.70-0.87)	109.5 (105.3-113.7)	0.75 (0.71-0.78)	215.0 (207.5-222.5)	0.77 (0.73-0.80)	340.4 (328.1-352.8)	0.63 (0.60-0.66)
2005	63.5 (58.4-68.6)	0.81 (0.73-0.90)	114.5 (110.3-118.7)	0.78 (0.74-0.82)	223.8 (216.4-231.3)	0.80 (0.76-0.83)	368.3 (355.7-380.9)	0.68 (0.65-0.71)
2006	68.9 (63.7-74.2)	0.88 (0.80-0.98)	118.2 (114.0-122.4)	0.81 (0.77-0.85)	230.4 (223.0-237.8)	0.82 (0.79-0.86)	383.4 (370.7-396.0)	0.71 (0.68-0.74)
2007	70.6 (65.3-75.9)	0.90 (0.82-1.00)	119.9 (115.7-124.2)	0.82 (0.78-0.86)	235.7 (228.3-243.2)	0.84 (0.81-0.88)	393.7 (381.0-406.4)	0.73 (0.70-0.76)
2008	72.2 (66.9-77.5)	0.92 (0.84-1.02)	122.4 (118.1-126.6)	0.83 (0.80-0.87)	240.0 (232.6-247.4)	0.86 (0.82-0.89)	401.3 (388.6-413.9)	0.74 (0.71-0.77)
2009	72.8 (67.5-78.0)	0.93 (0.84-1.03)	124.8 (120.6-129.1)	0.85 (0.81-0.89)	247.1 (239.6-254.5)	0.88 (0.85-0.92)	415.3 (402.6-428.0)	0.77 (0.74-0.80)
2010	72.6 (67.4-77.9)	0.93 (0.84-1.03)	129.8 (125.4-134.1)	0.88 (0.84-0.93)	251.2 (243.8-258.7)	0.90 (0.86-0.93)	434.0 (421.1-446.9)	0.80 (0.77-0.84)
2011	71.3 (66.2-76.5)	0.91 (0.83-1.01)	133.6 (129.2-138.1)	0.91 (0.87-0.95)	256.6 (249.0-264.1)	0.91 (0.88-0.95)	441.7 (428.8-454.6)	0.82 (0.79-0.85)
2012	73.5 (68.3-78.8)	0.94 (0.85-1.04)	135.7 (131.2-140.2)	0.92 (0.88-0.97)	259.4 (251.9-267.0)	0.93 (0.89-0.96)	454.7 (441.7-467.7)	0.84 (0.81-0.87)
2013	76.8 (71.5-82.1)	0.98 (0.89-1.08)	137.0 (132.5-141.5)	0.93 (0.89-0.98)	264.0 (256.4-271.6)	0.94 (0.90-0.98)	468.9 (455.9-481.9)	0.87 (0.84-0.90)
2014	75.9 (70.7-81.2)	0.97 (0.88-1.07)	140.5 (135.8-145.1)	0.96 (0.91-1.00)	269.7 (261.9-277.5)	0.96 (0.92-1.00)	482.4 (469.1-495.7)	0.89 (0.86-0.93)
2015	76.3 (71.1-81.6)	0.98 (0.89-1.08)	141.5 (136.8-146.2)	0.96 (0.92-1.01)	273.6 (265.8-281.5)	0.98 (0.94-1.02)	492.7 (479.3-506.1)	0.91 (0.88-0.95)
2016	75.3 (70.1-80.5)	0.96 (0.87-1.06)	143.5 (138.7-148.2)	0.98 (0.93-1.02)	274.7 (266.8-282.5)	0.98 (0.94-1.02)	508.2 (494.6-521.8)	0.94 (0.91-0.98)
2017	77.0 (71.7-82.3)	0.99 (0.89-1.09)	145.5 (140.7-150.3)	0.99 (0.95-1.04)	279.0 (271.0-286.9)	0.99 (0.96-1.04)	522.5 (508.7-536.3)	0.97 (0.93-1.00)
2018	76.9 (71.6-82.1)	0.98 (0.89-1.08)	145.0 (140.2-149.8)	0.99 (0.94-1.04)	278.9 (271.0-286.9)	0.99 (0.96-1.04)	530.5 (516.7-544.3)	0.98 (0.95-1.02)
2019	78.1 (72.8-83.4)	1	146.7 (141.9-151.5)	1	280.5 (272.5-288.4)	1	540.2 (526.3-554.0)	1

Note: All rates are per 100,000 persons and have been age standardised to CPRD population as of 1/1/2019
