# Supplementary material for: Prevalence and incidence of neuromuscular conditions in the UK between 2000 and 2019: A retrospective study using primary care data
Source: PLoS One. 2021 Dec 31;16(12):e0261983. doi: 10.1371/journal.pone.0261983 (PMC8719665; doi:10.1371/journal.pone.0261983)
Supplement: S13 Table — (PDF) [file pone.0261983.s013.pdf]

Table S13 – Age standardised prevalence rates 2000-19 for all neuromuscular disease in females by age, excluding Guillain-Barré syndrome codes not recorded in previous 5 years

Year	Females 0-14 years		Females 15-44 years		Females 45-64 years		Females 65+ years	
	Prevalence Rate (95%CI)	Rate Ratio (95%CI)	Prevalence Rate (95%CI)	Rate Ratio (95%CI)	Prevalence Rate (95%CI)	Rate Ratio (95%CI)	Prevalence Rate (95%CI)	Rate Ratio (95%CI)
2000	30.1 (25.5-34.6)	0.69 (0.58-0.82)	76.9 (72.6-81.1)	0.68 (0.63-0.72)	162.1 (154.1-170.0)	0.71 (0.67-0.75)	183.6 (174.3-192.9)	0.51 (0.48-0.54)
2001	29.3 (25.0-33.6)	0.67 (0.56-0.80)	79.7 (75.5-83.8)	0.70 (0.66-0.75)	166.1 (158.4-173.7)	0.72 (0.68-0.77)	193.1 (184.0-202.2)	0.54 (0.51-0.57)
2002	31.0 (26.8-35.2)	0.71 (0.60-0.83)	83.6 (79.6-87.6)	0.74 (0.69-0.78)	173.7 (166.2-181.1)	0.76 (0.72-0.80)	207.0 (197.9-216.0)	0.58 (0.55-0.61)
2003	33.5 (29.3-37.7)	0.77 (0.65-0.89)	86.3 (82.3-90.2)	0.76 (0.72-0.81)	176.9 (169.7-184.2)	0.77 (0.73-0.81)	214.7 (205.8-223.7)	0.60 (0.57-0.63)
2004	34.3 (30.3-38.4)	0.78 (0.68-0.91)	91.7 (87.8-95.7)	0.81 (0.76-0.86)	184.6 (177.5-191.7)	0.80 (0.77-0.85)	228.5 (219.6-237.4)	0.64 (0.61-0.67)
2005	37.1 (33.0-41.2)	0.85 (0.73-0.98)	94.9 (91.0-98.8)	0.84 (0.79-0.88)	194.3 (187.2-201.4)	0.85 (0.81-0.89)	240.7 (231.7-249.7)	0.67 (0.64-0.70)
2006	38.6 (34.4-42.7)	0.88 (0.77-1.02)	97.5 (93.6-101.4)	0.86 (0.81-0.91)	199.4 (192.4-206.5)	0.87 (0.83-0.91)	251.8 (242.7-260.9)	0.70 (0.67-0.74)
2007	38.7 (34.6-42.8)	0.89 (0.77-1.02)	99.6 (95.7-103.6)	0.88 (0.83-0.93)	201.8 (194.8-208.8)	0.88 (0.84-0.92)	264.6 (255.4-273.9)	0.74 (0.70-0.77)
2008	39.6 (35.5-43.7)	0.91 (0.79-1.04)	99.3 (95.4-103.2)	0.87 (0.83-0.92)	206.9 (199.9-213.9)	0.90 (0.86-0.94)	270.9 (261.6-280.2)	0.76 (0.72-0.79)
2009	41.2 (37.1-45.4)	0.94 (0.82-1.08)	101.4 (97.5-105.4)	0.89 (0.85-0.94)	211.4 (204.4-218.4)	0.92 (0.88-0.96)	285.0 (275.6-294.5)	0.79 (0.76-0.83)
2010	43.4 (39.2-47.6)	0.99 (0.87-1.14)	102.1 (98.1-106.0)	0.90 (0.85-0.95)	213.6 (206.6-220.5)	0.93 (0.89-0.97)	296.3 (286.7-305.9)	0.83 (0.79-0.86)
2011	42.9 (38.7-47.0)	0.98 (0.86-1.12)	102.4 (114.4-106.3)	0.90 (0.85-0.95)	215.7 (208.7-222.7)	0.94 (0.90-0.98)	305.6 (295.9-315.3)	0.85 (0.82-0.89)
2012	44.3 (40.1-48.5)	1.01 (0.89-1.16)	103.3 (99.3-107.3)	0.91 (0.86-0.96)	214.5 (207.5-221.5)	0.94 (0.89-0.98)	315.7 (305.9-325.5)	0.88 (0.84-0.92)
2013	45.7 (41.5-49.9)	1.04 (0.92-1.19)	102.9 (99.0-106.9)	0.91 (0.86-0.96)	215.7 (208.8-222.7)	0.94 (0.90-0.98)	324.2 (314.3-334.0)	0.90 (0.87-0.94)
2014	45.9 (41.7-50.1)	1.05 (0.92-1.20)	106.3 (102.2-110.4)	0.94 (0.89-0.99)	218.9 (211.8-226.1)	0.95 (0.91-1.00)	332.7 (322.7-342.8)	0.93 (0.89-0.97)
2015	45.8 (41.6-49.9)	1.05 (0.92-1.19)	109.8 (105.6-114.0)	0.97 (0.92-1.02)	220.0 (212.8-227.1)	0.96 (0.92-1.00)	336.2 (326.1-346.3)	0.94 (0.90-0.98)
2016	43.4 (39.3-47.4)	0.99 (0.87-1.13)	110.3 (106.1-114.5)	0.97 (0.92-1.03)	224.6 (217.3-231.8)	0.98 (0.94-1.02)	339.0 (328.8-349.2)	0.95 (0.91-0.99)
2017	44.1 (40.0-48.2)	1.01 (0.88-1.15)	110.7 (106.4-114.9)	0.97 (0.92-1.03)	224.2 (217.0-231.5)	0.98 (0.93-1.02)	343.3 (333.0-353.5)	0.96 (0.92-1.00)
2018	43.8 (39.7-47.9)	1.00 (0.88-1.14)	110.8 (106.6-115.0)	0.98 (0.93-1.03)	226.8 (219.6-234.1)	0.99 (0.95-1.03)	351.0 (340.7-361.4)	0.98 (0.94-1.02)
2019	43.7 (39.7-47.8)	1	113.5 (109.3-117.8)	1	229.4 (222.1-236.7)	1	358.6 (348.2-369.1)	1

Note: All rates are per 100,000 persons and have been age standardised to CPRD population as of 1/1/2019
