# Supplementary material for: Prevalence and incidence of neuromuscular conditions in the UK between 2000 and 2019: A retrospective study using primary care data
Source: PLoS One. 2021 Dec 31;16(12):e0261983. doi: 10.1371/journal.pone.0261983 (PMC8719665; doi:10.1371/journal.pone.0261983)
Supplement: S14 Table — (PDF) [file pone.0261983.s014.pdf]

Table S14 – Age standardised prevalence rates 2000-19 for all neuromuscular disease in males by age, excluding Guillain-Barré syndrome codes not recorded in previous 5 years

Year	Males 0-14 years		Males 15-44 years		Males 45-64 years		Males 65+ years	
	Prevalence Rate (95%CI)	Rate Ratio (95%CI)	Prevalence Rate (95%CI)	Rate Ratio (95%CI)	Prevalence Rate (95%CI)	Rate Ratio (95%CI)	Prevalence Rate (95%CI)	Rate Ratio (95%CI)
2000	57.2 (51.3-63.1)	0.74 (0.66-0.84)	79.2 (74.9-83.4)	0.60 (0.57-0.64)	159.5 (151.7-167.2)	0.67 (0.63-0.71)	237.6 (225.4-249.7)	0.52 (0.49-0.55)
2001	59.7 (54.0-65.4)	0.77 (0.69-0.87)	85.1 (80.9-89.3)	0.65 (0.61-0.69)	162.8 (155.3-170.3)	0.68 (0.65-0.72)	248.5 (236.7-260.4)	0.54 (0.51-0.57)
2002	58.8 (53.4-64.2)	0.76 (0.68-0.86)	90.3 (86.2-94.5)	0.69 (0.65-0.73)	167.3 (160.1-174.5)	0.70 (0.67-0.74)	262.7 (251.0-274.4)	0.57 (0.54-0.60)
2003	59.3 (54.1-64.6)	0.77 (0.69-0.86)	93.6 (89.6-97.7)	0.71 (0.67-0.75)	173.5 (166.5-180.6)	0.73 (0.69-0.77)	279.4 (267.7-291.1)	0.61 (0.58-0.64)
2004	59.4 (54.4-64.5)	0.77 (0.69-0.86)	97.0 (93.1-101.0)	0.74 (0.70-0.78)	182.3 (175.4-189.2)	0.77 (0.73-0.80)	290.4 (279.0-301.8)	0.63 (0.60-0.66)
2005	62.1 (57.0-67.2)	0.81 (0.72-0.90)	101.5 (97.6-105.5)	0.77 (0.73-0.81)	189.9 (183.1-196.8)	0.80 (0.76-0.84)	316.6 (305.0-328.3)	0.69 (0.66-0.72)
2006	67.8 (62.6-73.0)	0.88 (0.79-0.97)	104.5 (100.6-108.5)	0.80 (0.76-0.84)	196.0 (189.2-202.9)	0.82 (0.79-0.86)	327.8 (316.1-339.6)	0.71 (0.68-0.74)
2007	68.9 (63.6-74.1)	0.89 (0.81-0.99)	106.0 (102.0-110.0)	0.81 (0.77-0.85)	200.4 (193.6-207.2)	0.84 (0.80-0.88)	336.9 (325.1-348.6)	0.73 (0.70-0.76)
2008	70.3 (65.1-75.6)	0.91 (0.82-1.01)	108.0 (104.0-111.9)	0.82 (0.78-0.86)	202.4 (195.6-209.2)	0.85 (0.81-0.89)	342.4 (330.7-354.0)	0.74 (0.71-0.78)
2009	71.3 (66.1-76.6)	0.93 (0.84-1.02)	110.5 (106.5-114.6)	0.84 (0.80-0.88)	207.8 (200.9-214.6)	0.87 (0.83-0.91)	354.7 (342.9-366.4)	0.77 (0.74-0.80)
2010	70.6 (65.5-75.8)	0.92 (0.83-1.01)	114.9 (110.8-119.0)	0.87 (0.83-0.92)	211.2 (204.4-218.1)	0.89 (0.85-0.93)	371.2 (359.3-383.1)	0.80 (0.77-0.84)
2011	69.2 (64.1-74.3)	0.90 (0.81-0.99)	118.5 (114.4-122.7)	0.90 (0.86-0.95)	215.2 (208.3-222.0)	0.90 (0.86-0.94)	377.0 (365.1-388.9)	0.82 (0.78-0.85)
2012	71.1 (66.0-76.3)	0.92 (0.84-1.02)	120.4 (116.2-124.6)	0.92 (0.87-0.96)	218.2 (211.2-225.1)	0.92 (0.88-0.96)	388.3 (376.3-400.3)	0.84 (0.81-0.88)
2013	74.8 (69.6-80.1)	0.97 (0.88-1.07)	121.5 (117.3-125.8)	0.92 (0.88-0.97)	223.1 (216.1-230.1)	0.94 (0.90-0.98)	399.0 (386.9-411.0)	0.87 (0.83-0.90)
2014	74.2 (69.0-79.4)	0.96 (0.87-1.06)	124.9 (120.5-129.3)	0.95 (0.90-1.00)	227.5 (220.3-234.7)	0.95 (0.91-1.00)	409.5 (397.2-421.8)	0.89 (0.85-0.93)
2015	75.0 (69.7-80.2)	0.97 (0.88-1.07)	125.9 (121.5-130.3)	0.96 (0.91-1.01)	230.9 (223.7-238.1)	0.97 (0.93-1.01)	417.8 (405.4-430.1)	0.91 (0.87-0.94)
2016	74.0 (68.8-79.1)	0.96 (0.87-1.06)	127.5 (123.0-132.0)	0.97 (0.92-1.02)	233.2 (225.9-240.4)	0.98 (0.94-1.02)	432.4 (419.8-444.9)	0.94 (0.90-0.98)
2017	75.5 (70.3-80.7)	0.98 (0.89-1.08)	129.8 (125.2-134.3)	0.99 (0.94-1.04)	237.0 (229.6-244.3)	0.99 (0.95-1.04)	444.0 (431.3-456.7)	0.96 (0.93-1.00)
2018	75.4 (70.2-80.6)	0.98 (0.89-1.08)	129.5 (125.0-134.0)	0.99 (0.94-1.04)	237.4 (230.1-244.7)	1.00 (0.95-1.04)	451.7 (438.9-464.4)	0.98 (0.94-1.02)
2019	77.1 (71.8-82.4)	1	131.4 (126.8-135.9)	1	238.2 (230.9-245.6)	1	461.1 (448.3-474.0)	1

Note: All rates are per 100,000 persons and have been age standardised to CPRD population as of 1/1/2019
