# Supplementary material for: Prevalence and incidence of neuromuscular conditions in the UK between 2000 and 2019: A retrospective study using primary care data
Source: PLoS One. 2021 Dec 31;16(12):e0261983. doi: 10.1371/journal.pone.0261983 (PMC8719665; doi:10.1371/journal.pone.0261983)
Supplement: S15 Table — (PDF) [file pone.0261983.s015.pdf]

Table S15 – Age standardised incidence rates 2000-19 for all neuromuscular disease in females by age

Year	Females 0-14 years		Females 15-44 years		Females 45-64 years		Females 65+ years	
	Incidence Rate (95%CI)	Rate Ratio (95%CI)	Incidence Rate (95%CI)	Rate Ratio (95%CI)	Incidence Rate (95%CI)	Rate Ratio (95%CI)	Incidence Rate (95%CI)	Rate Ratio (95%CI)
2000	3.2 (1.8-4.6)	0.60 (0.36-1.03)	7.5 (6.2-8.9)	1.14 (0.89-1.45)	16.1 (13.6-18.6)	1.17 (0.95-1.45)	19.7 (16.7-22.8)	0.72 (0.60-0.88)
2001	4.2 (2.7-5.7)	0.80 (0.50-1.28)	7.7 (6.4-8.9)	1.15 (0.91-1.46)	17.7 (15.2-20.1)	1.28 (1.05-1.57)	22.8 (19.6-25.9)	0.83 (0.70-1.00)
2002	6.0 (4.2-7.7)	1.13 (0.75-1.72)	7.0 (5.8-8.1)	1.05 (0.83-1.33)	15.8 (13.6-18.1)	1.15 (0.94-1.41)	22.0 (19.0-24.9)	0.80 (0.67-0.96)
2003	5.9 (4.2-7.5)	1.12 (0.74-1.68)	8.3 (7.0-9.5)	1.25 (0.99-1.56)	17.2 (15.0-19.4)	1.25 (1.03-1.52)	26.5 (23.3-29.6)	0.97 (0.82-1.14)
2004	5.2 (3.7-6.8)	1.00 (0.66-1.51)	7.7 (6.6-8.8)	1.16 (0.93-1.45)	17.2 (15.0-19.4)	1.25 (1.03-1.51)	25.7 (22.6-28.7)	0.94 (0.80-1.11)
2005	5.1 (3.6-6.6)	0.97 (0.64-1.47)	7.6 (6.5-8.7)	1.15 (0.92-1.44)	15.8 (13.8-17.8)	1.15 (0.95-1.39)	26.5 (23.5-29.5)	0.97 (0.83-1.14)
2006	5.6 (4.1-7.0)	1.06 (0.71-1.58)	7.2 (6.2-8.3)	1.09 (0.87-1.36)	13.9 (12.0-15.7)	1.01 (0.83-1.23)	27.5 (24.4-30.5)	1.01 (0.86-1.18)
2007	6.6 (5.0-8.2)	1.26 (0.86-1.85)	5.5 (4.6-6.5)	0.84 (0.66-1.06)	15.7 (13.8-17.7)	1.14 (0.95-1.38)	23.8 (21.0-26.6)	0.87 (0.74-1.03)
2008	4.2 (2.9-5.5)	0.80 (0.53-1.23)	7.3 (6.3-8.4)	1.11 (0.88-1.38)	14.9 (13.0-16.7)	1.08 (0.89-1.31)	28.4 (25.4-31.5)	1.04 (0.89-1.22)
2009	5.2 (3.8-6.6)	0.99 (0.66-1.48)	5.9 (5.0-6.8)	0.89 (0.71-1.13)	15.5 (13.6-17.4)	1.12 (0.93-1.36)	26.0 (23.1-28.9)	0.95 (0.81-1.12)
2010	4.3 (3.0-5.5)	0.81 (0.53-1.24)	6.8 (5.8-7.8)	1.03 (0.82-1.29)	14.5 (12.7-16.4)	1.06 (0.87-1.28)	27.1 (24.1-30.0)	0.99 (0.84-1.16)
2011	4.6 (3.3-5.9)	0.88 (0.59-1.32)	5.6 (4.7-6.5)	0.84 (0.66-1.07)	13.6 (11.8-15.4)	0.99 (0.81-1.20)	25.8 (22.9-28.6)	0.94 (0.80-1.11)
2012	5.0 (3.7-6.3)	0.95 (0.64-1.41)	6.8 (5.8-7.8)	1.03 (0.82-1.29)	13.7 (11.9-15.5)	1.00 (0.82-1.21)	28.1 (25.2-31.1)	1.03 (0.88-1.21)
2013	6.0 (4.5-7.5)	1.15 (0.78-1.69)	6.9 (5.9-8.0)	1.04 (0.83-1.31)	14.1 (12.3-15.9)	1.02 (0.84-1.24)	27.7 (24.7-30.6)	1.01 (0.86-1.19)
2014	4.8 (3.5-6.1)	0.92 (0.61-1.37)	7.0 (5.9-8.0)	1.05 (0.84-1.32)	13.2 (11.4-15.0)	0.96 (0.79-1.17)	25.7 (22.8-28.5)	0.94 (0.80-1.11)
2015	4.2 (3.0-5.4)	0.79 (0.52-1.20)	6.3 (5.3-7.3)	0.95 (0.75-1.20)	15.0 (13.1-16.9)	1.09 (0.90-1.32)	25.8 (22.9-28.6)	0.94 (0.80-1.11)
2016	4.9 (3.5-6.2)	0.92 (0.62-1.38)	7.5 (6.4-8.6)	1.12 (0.90-1.41)	13.4 (11.6-15.1)	0.97 (0.80-1.18)	24.9 (22.1-27.7)	0.91 (0.78-1.07)
2017	6.3 (4.8-7.8)	1.20 (0.82-1.75)	6.4 (5.4-7.4)	0.96 (0.76-1.21)	14.3 (12.5-16.2)	1.04 (0.86-1.26)	28.1 (25.1-31.1)	1.03 (0.88-1.21)
2018	4.4 (3.1-5.6)	0.83 (0.55-1.25)	7.7 (6.6-8.8)	1.16 (0.93-1.45)	14.0 (12.2-15.9)	1.02 (0.84-1.24)	29.1 (26.1-32.2)	1.07 (0.91-1.25)
2019	5.3 (3.7-6.8)	1	6.6 (5.5-7.8)	1	13.8 (11.8-15.7)	1	27.3 (24.1-30.5)	1

Note: All rates are per 100,000 person years and have been age standardised to CPRD population as of 1/1/2019
