# Supplementary material for: Prevalence and incidence of neuromuscular conditions in the UK between 2000 and 2019: A retrospective study using primary care data
Source: PLoS One. 2021 Dec 31;16(12):e0261983. doi: 10.1371/journal.pone.0261983 (PMC8719665; doi:10.1371/journal.pone.0261983)
Supplement: S16 Table — (PDF) [file pone.0261983.s016.pdf]

Table S16 – Age standardised incidence rates 2000-19 for all neuromuscular disease in males by age

Year	Males 0-14 years		Males 15-44 years		Males 45-64 years		Males 65+ years	
	Incidence Rate (95%CI)	Rate Ratio (95%CI)	Incidence Rate (95%CI)	Rate Ratio (95%CI)	Incidence Rate (95%CI)	Rate Ratio (95%CI)	Incidence Rate (95%CI)	Rate Ratio (95%CI)
2000	7.8 (53.3-65.2)	1.00 (0.70-1.44)	7.3 (87.0-96.2)	1.03 (0.81-1.30)	18.4 (178.3-195.2)	1.14 (0.94-1.38)	33.9 (29.3-38.5)	0.91 (0.76-1.08)
2001	7.5 (55.5-67.1)	0.97 (0.68-1.38)	7.1 (93.1-102.1)	0.99 (0.78-1.26)	19.5 (182.8-198.9)	1.21 (1.01-1.46)	35.8 (31.3-40.3)	0.96 (0.81-1.13)
2002	6.7 (54.8-65.7)	0.86 (0.60-1.23)	6.9 (98.3-107.1)	0.97 (0.77-1.23)	18.5 (188.9-204.4)	1.15 (0.96-1.38)	41.6 (36.9-46.3)	1.11 (0.95-1.30)
2003	8.5 (55.2-65.9)	1.09 (0.79-1.51)	7.3 (101.9-110.5)	1.03 (0.82-1.29)	20.6 (196.7-212.0)	1.28 (1.08-1.53)	33.2 (29.2-37.2)	0.89 (0.75-1.04)
2004	8.1 (56.0-66.2)	1.04 (0.75-1.44)	6.7 (105.3-113.7)	0.94 (0.75-1.18)	19.6 (207.5-222.5)	1.22 (1.02-1.45)	39.2 (35.0-43.4)	1.05 (0.90-1.22)
2005	8.5 (58.4-68.6)	1.10 (0.80-1.51)	6.9 (110.3-118.7)	0.97 (0.78-1.21)	18.5 (216.4-231.3)	1.15 (0.97-1.37)	38.2 (34.1-42.3)	1.02 (0.88-1.19)
2006	8.2 (63.7-74.2)	1.05 (0.77-1.45)	6.3 (114.0-122.4)	0.89 (0.71-1.11)	17.3 (223.0-237.8)	1.08 (0.90-1.28)	37.2 (33.2-41.2)	0.99 (0.85-1.16)
2007	8.3 (65.3-75.9)	1.07 (0.78-1.47)	6.0 (115.7-124.2)	0.84 (0.67-1.05)	18.4 (228.3-243.2)	1.14 (0.96-1.36)	34.9 (31.1-38.7)	0.93 (0.80-1.09)
2008	7.6 (66.9-77.5)	0.98 (0.71-1.35)	6.2 (118.1-126.6)	0.87 (0.69-1.09)	19.4 (232.6-247.4)	1.20 (1.02-1.43)	34.9 (31.1-38.7)	0.93 (0.80-1.09)
2009	7.7 (67.5-78.0)	0.99 (0.72-1.37)	6.3 (120.6-129.1)	0.89 (0.71-1.11)	17.5 (239.6-254.5)	1.09 (0.91-1.29)	39.3 (35.3-43.3)	1.05 (0.90-1.22)
2010	8.5 (67.4-77.9)	1.10 (0.81-1.50)	6.7 (125.4-134.1)	0.94 (0.76-1.17)	17.8 (243.8-258.7)	1.11 (0.93-1.31)	37.1 (33.3-41.0)	0.99 (0.85-1.15)
2011	6.4 (66.2-76.5)	0.82 (0.59-1.14)	6.2 (129.2-138.1)	0.87 (0.70-1.09)	17.4 (249.0-264.1)	1.08 (0.91-1.28)	36.9 (33.1-40.7)	0.98 (0.85-1.14)
2012	8.9 (68.3-78.8)	1.15 (0.84-1.56)	7.3 (131.2-140.2)	1.02 (0.82-1.27)	18.0 (251.9-267.0)	1.12 (0.94-1.33)	39.2 (35.3-43.1)	1.05 (0.90-1.21)
2013	7.0 (71.5-82.1)	0.91 (0.66-1.25)	7.0 (132.5-141.5)	0.99 (0.79-1.23)	16.8 (256.4-271.6)	1.04 (0.87-1.24)	39.9 (36.0-43.8)	1.07 (0.92-1.23)
2014	7.5 (70.7-81.2)	0.96 (0.70-1.32)	6.4 (135.8-145.1)	0.90 (0.72-1.13)	16.9 (261.9-277.5)	1.05 (0.88-1.25)	38.2 (34.4-42.1)	1.02 (0.88-1.18)
2015	9.1 (71.1-81.6)	1.17 (0.86-1.59)	6.8 (136.8-146.2)	0.96 (0.77-1.19)	19.1 (265.8-281.5)	1.19 (1.00-1.41)	45.5 (41.3-49.6)	1.21 (1.05-1.40)
2016	8.3 (70.1-80.5)	1.07 (0.78-1.46)	7.2 (138.7-148.2)	1.01 (0.81-1.26)	18.5 (266.8-282.5)	1.15 (0.97-1.37)	42.4 (38.4-46.4)	1.13 (0.98-1.31)
2017	7.5 (71.7-82.3)	0.96 (0.70-1.32)	6.3 (140.7-150.3)	0.89 (0.71-1.11)	18.6 (271.0-286.9)	1.16 (0.98-1.37)	39.1 (35.3-43.0)	1.04 (0.90-1.21)
2018	8.4 (71.6-82.1)	1.08 (0.79-1.47)	7.2 (140.2-149.8)	1.01 (0.81-1.26)	19.1 (271.0-286.9)	1.19 (1.00-1.41)	39.6 (35.8-43.4)	1.06 (0.91-1.22)
2019	7.8 (72.8-83.4)	1	7.1 (141.9-151.5)	1	16.1 (272.5-288.4)	1	37.5 (33.4-41.5)	1

Note: All rates are per 100,000 person years and have been age standardised to CPRD population as of 1/1/2019
