# Supplementary material for: Prevalence and incidence of neuromuscular conditions in the UK between 2000 and 2019: A retrospective study using primary care data
Source: PLoS One. 2021 Dec 31;16(12):e0261983. doi: 10.1371/journal.pone.0261983 (PMC8719665; doi:10.1371/journal.pone.0261983)
Supplement: S17 Table — (PDF) [file pone.0261983.s017.pdf]

Table S17 – Age standardised prevalence rates 2000-19 for selected conditions

Year	Inflammatory myopathies	Muscular dystrophies	Charcot-Marie Tooth disease	Guillain-Barré syndrome		Myasthenia gravis	Motor neurone disease
				Lifetime	Code in last 5 years		
	Prevalence Rate (95%CI)	Prevalence Rate (95%CI)	Prevalence Rate (95%CI)	Prevalence Rate (95%CI)	Prevalence Rate (95%CI)	Prevalence Rate (95%CI)	Prevalence Rate (95%CI)
2000	16.0 (15.1-16.9)	23.7 (22.6-24.8)	12.3 (11.5-13.1)	25.6 (24.5-26.7)	7.4 (6.8-8.0)	18.6 (17.7-19.6)	11.5 (10.8-12.3)
2001	16.2 (15.3-17.0)	25.0 (24.0-26.1)	13.1 (12.3-13.8)	26.5 (25.5-27.6)	7.8 (7.2-8.4)	19.4 (18.4-20.3)	11.7 (10.9-12.4)
2002	16.8 (16.0-17.7)	25.6 (24.6-26.6)	14.0 (13.3-14.8)	27.6 (26.6-28.7)	8.1 (7.5-8.7)	20.5 (19.6-21.4)	11.8 (11.1-12.5)
2003	17.7 (16.9-18.5)	26.5 (25.5-27.5)	14.6 (13.8-15.3)	28.7 (27.7-29.8)	8.3 (7.7-8.9)	21.5 (20.6-22.4)	12.1 (11.4-12.8)
2004	18.1 (17.4-18.9)	27.3 (26.3-28.2)	15.8 (15.0-16.5)	29.9 (28.9-30.9)	8.8 (8.2-9.3)	22.9 (22.0-23.8)	12.1 (11.5-12.8)
2005	19.4 (18.6-20.2)	28.5 (27.5-29.4)	17.1 (16.4-17.9)	31.0 (30.0-32.0)	9.0 (8.5-9.6)	23.7 (22.8-24.6)	12.5 (11.9-13.2)
2006	19.8 (19.0-20.6)	29.3 (28.3-30.3)	18.5 (17.7-19.2)	32.1 (31.1-33.2)	9.1 (8.6-9.7)	24.6 (23.7-25.5)	12.6 (11.9-13.2)
2007	20.3 (19.5-21.1)	29.8 (28.9-30.8)	20.0 (19.2-20.8)	32.9 (31.9-33.9)	9.2 (8.6-9.7)	25.2 (24.3-26.1)	12.3 (11.7-13.0)
2008	21.0 (20.2-21.8)	29.8 (28.8-30.8)	20.9 (20.1-21.7)	33.4 (32.4-34.5)	9.0 (8.5-9.6)	25.5 (24.6-26.4)	12.0 (11.4-12.6)
2009	21.7 (20.8-22.5)	30.0 (29.1-31.0)	21.9 (21.1-22.7)	34.4 (33.3-35.4)	9.3 (8.8-9.8)	26.7 (25.8-27.6)	12.3 (11.6-12.9)
2010	22.1 (21.3-22.9)	30.3 (29.4-31.3)	22.9 (22.0-23.7)	35.4 (34.3-36.4)	9.5 (9.0-10.0)	27.6 (26.7-28.5)	12.6 (11.9-13.2)
2011	22.7 (21.9-23.5)	30.3 (29.4-31.3)	23.8 (23.0-24.7)	35.9 (34.9-36.9)	9.5 (9.0-10.0)	27.9 (27.0-28.9)	12.6 (11.9-13.2)
2012	22.9 (22.1-23.7)	30.7 (29.7-31.6)	24.7 (23.9-25.6)	36.5 (35.4-37.5)	9.6 (9.1-10.2)	28.5 (27.6-29.4)	12.8 (12.1-13.4)
2013	23.2 (22.4-24.1)	30.7 (29.8-31.7)	25.2 (24.3-26.0)	37.3 (36.3-38.4)	10.0 (9.4-10.5)	29.2 (28.2-30.1)	12.8 (12.2-13.4)
2014	23.8 (23.0-24.7)	30.6 (29.6-31.6)	26.1 (25.3-27.0)	38.4 (37.3-39.4)	10.1 (9.5-10.7)	30.0 (29.1-31.0)	12.6 (12.0-13.3)
2015	24.3 (23.4-25.1)	30.3 (29.3-31.2)	27.0 (26.1-27.9)	38.6 (37.5-39.7)	9.9 (9.3-10.4)	30.8 (29.9-31.8)	12.3 (11.6-12.9)
2016	24.5 (23.6-25.4)	29.9 (29.0-30.9)	27.5 (26.6-28.5)	38.9 (37.8-40.0)	10.1 (9.5-10.6)	31.4 (30.5-32.4)	12.7 (12.0-13.3)
2017	24.8 (23.9-25.7)	29.4 (28.4-30.3)	28.1 (27.2-29.0)	39.3 (38.2-40.4)	10.3 (9.7-10.9)	32.5 (31.5-33.6)	12.7 (12.1-13.3)
2018	25.0 (24.1-25.9)	29.3 (28.4-30.2)	28.7 (27.8-29.6)	39.7 (38.6-40.8)	10.5 (9.9-11.0)	32.9 (31.9-33.9)	12.3 (11.7-12.9)
2019	25.0 (24.1-25.8)	29.5 (28.5-30.4)	29.5 (28.5-30.4)	40.1 (39.0-41.2)	10.5 (9.9-11.1)	33.7 (32.7-34.7)	12.6 (11.9-13.2)

Note: All rates are per 100,000 years and have been age standardised to CPRD population as of 1/1/2019
