# Supplementary material for: Prevalence and incidence of neuromuscular conditions in the UK between 2000 and 2019: A retrospective study using primary care data
Source: PLoS One. 2021 Dec 31;16(12):e0261983. doi: 10.1371/journal.pone.0261983 (PMC8719665; doi:10.1371/journal.pone.0261983)
Supplement: S18 Table — (PDF) [file pone.0261983.s018.pdf]

Table S18 – Age standardised incidence rates 2000-19 for selected conditions

Year	Inflammatory myopathies	Muscular dystrophies	Charcot-Marie Tooth disease	Guillain-Barré syndrome	Myasthenia gravis	Motor neurone disease
	Incidence Rate (95%CI)	Incidence Rate (95%CI)	Incidence Rate (95%CI)	Incidence Rate (95%CI)	Incidence Rate (95%CI)	Incidence Rate (95%CI)
2000	1.3 (1.1-1.6)	1.9 (1.6-2.2)	0.9 (0.7-1.1)	1.8 (1.5-2.1)	1.7 (1.4-2.0)	3.2 (2.8-3.6)
2001	1.6 (1.4-1.9)	1.7 (1.4-2.0)	1.1 (0.9-1.3)	1.6 (1.4-1.9)	2.0 (1.7-2.3)	3.0 (2.7-3.4)
2002	1.6 (1.4-1.9)	1.7 (1.4-1.9)	1.0 (0.8-1.2)	1.8 (1.6-2.1)	2.0 (1.7-2.3)	3.4 (3.0-3.8)
2003	1.4 (1.1-1.6)	2.0 (1.7-2.2)	1.4 (1.1-1.6)	1.9 (1.6-2.2)	2.1 (1.9-2.4)	3.0 (2.7-3.3)
2004	1.9 (1.6-2.1)	1.8 (1.6-2.1)	1.6 (1.4-1.9)	1.5 (1.3-1.7)	1.7 (1.4-1.9)	3.2 (2.9-3.5)
2005	1.4 (1.2-1.7)	1.6 (1.4-1.9)	1.5 (1.3-1.7)	1.5 (1.3-1.8)	2.0 (1.8-2.3)	3.5 (3.2-3.9)
2006	1.4 (1.2-1.7)	1.7 (1.4-1.9)	1.7 (1.4-1.9)	1.5 (1.3-1.7)	2.1 (1.9-2.4)	3.2 (2.8-3.5)
2007	1.6 (1.4-1.8)	1.2 (1.0-1.4)	1.8 (1.5-2.0)	1.5 (1.3-1.7)	1.6 (1.4-1.9)	3.1 (2.7-3.4)
2008	1.3 (1.1-1.5)	1.4 (1.2-1.7)	1.5 (1.2-1.7)	1.9 (1.6-2.1)	2.4 (2.2-2.7)	3.1 (2.7-3.4)
2009	1.3 (1.1-1.5)	1.3 (1.1-1.5)	1.3 (1.1-1.5)	1.8 (1.6-2.1)	2.1 (1.9-2.4)	3.4 (3.1-3.8)
2010	1.4 (1.2-1.6)	1.3 (1.1-1.5)	1.4 (1.2-1.6)	1.6 (1.4-1.8)	2.0 (1.7-2.2)	3.7 (3.4-4.1)
2011	1.1 (0.9-1.3)	1.5 (1.3-1.7)	1.3 (1.1-1.5)	1.7 (1.4-1.9)	2.0 (1.8-2.3)	3.3 (3.0-3.7)
2012	1.3 (1.1-1.5)	1.4 (1.2-1.7)	1.2 (1.0-1.4)	1.7 (1.5-2.0)	2.3 (2.0-2.5)	3.6 (3.2-3.9)
2013	1.1 (0.9-1.3)	1.0 (0.8-1.2)	1.4 (1.2-1.6)	1.8 (1.6-2.0)	2.2 (1.9-2.5)	3.7 (3.4-4.1)
2014	1.1 (0.9-1.3)	1.0 (0.8-1.1)	1.3 (1.1-1.5)	1.5 (1.3-1.7)	2.5 (2.2-2.8)	3.4 (3.0-3.7)
2015	1.3 (1.1-1.5)	1.1 (0.9-1.3)	1.4 (1.2-1.6)	1.7 (1.5-2.0)	2.4 (2.1-2.7)	4.0 (3.6-4.3)
2016	1.3 (1.1-1.5)	1.1 (0.9-1.3)	1.5 (1.3-1.7)	1.8 (1.5-2.0)	2.5 (2.2-2.8)	3.5 (3.2-3.9)
2017	1.4 (1.2-1.6)	1.1 (0.9-1.3)	1.6 (1.4-1.9)	1.8 (1.5-2.0)	2.3 (2.1-2.6)	3.4 (3.0-3.7)
2018	1.1 (1.0-1.3)	1.1 (0.9-1.3)	1.4 (1.2-1.6)	1.9 (1.6-2.1)	2.9 (2.6-3.2)	3.6 (3.2-3.9)
2019	1.4 (1.2-1.6)	1.3 (1.1-1.5)	1.3 (1.1-1.5)	1.5 (1.2-1.7)	2.5 (2.2-2.8)	3.2 (2.8-3.5)

Note: All rates are per 100,000 person years have been age standardised to CPRD population as of 1/1/2019
