# Supplementary material for: Prevalence and incidence of neuromuscular conditions in the UK between 2000 and 2019: A retrospective study using primary care data
Source: PLoS One. 2021 Dec 31;16(12):e0261983. doi: 10.1371/journal.pone.0261983 (PMC8719665; doi:10.1371/journal.pone.0261983)
Supplement: S19 Table — (PDF) [file pone.0261983.s019.pdf]

Table S19 – Age standardised prevalence rates 2000-19 for selected conditions, ages 0-44 only

Year	Inflammatory myopathies		Muscular dystrophies		Charcot-Marie Tooth disease		Guillain-Barré syndrome				Myasthenia gravis		Motor neurone disease	
							Lifetime		Code in last 5 years					
	0-14y	15-44y	0-14y	15-44y	0-14y	15-44y	0-14y	15-44y	0-14y	15-44y	0-14y	15-44y	0-14y	15-44y
2000	1.9	8.7	19.6	23.0	4.8	10.4	4.5	17.9	2.5	4.8	1.3	8.4	1.3	2.4
2001	1.9	8.8	19.7	24.8	5.4	11.2	3.7	18.1	1.8	5.1	1.4	8.8	1.6	2.5
2002	1.9	8.7	19.1	25.7	5.1	12.5	3.7	18.7	1.6	5.7	1.5	9.1	1.6	2.7
2003	1.7	9.2	18.5	26.5	5.5	13.1	3.7	19.0	1.8	5.7	1.6	9.6	1.7	2.8
2004	1.9	9.3	18.0	27.1	5.3	14.2	3.9	19.1	1.9	6.0	1.3	9.8	1.5	2.9
2005	2.2	9.9	18.4	27.9	6.3	14.7	3.5	19.9	2.0	6.2	1.4	9.7	1.2	3.3
2006	2.6	10.0	19.5	28.2	5.6	15.6	3.8	20.5	2.5	6.2	1.2	9.7	1.4	3.7
2007	2.4	10.3	18.8	28.9	5.5	16.7	4.0	20.6	2.4	6.2	1.3	9.6	1.7	3.5
2008	2.8	10.9	18.7	28.3	5.8	17.3	4.2	20.0	2.6	5.7	1.3	9.8	1.6	3.6
2009	2.9	11.1	18.8	28.3	5.8	17.7	3.9	20.3	2.5	6.1	1.4	10.2	1.6	3.5
2010	3.0	11.3	18.9	28.8	5.7	18.3	4.0	20.9	2.2	6.4	1.3	10.7	1.5	3.4
2011	2.8	11.9	17.8	29.0	5.6	19.2	3.7	21.0	1.9	6.4	1.1	10.7	1.3	3.5
2012	2.8	12.5	18.0	29.2	6.3	20.2	4.5	20.4	2.4	5.9	1.0	10.4	1.4	3.4
2013	3.3	12.6	18.8	28.7	6.9	20.3	4.1	21.2	2.3	6.3	1.2	10.4	1.2	3.4
2014	3.2	12.8	17.7	28.6	7.2	21.2	3.7	21.7	2.2	6.5	1.3	10.9	1.4	3.4
2015	3.4	13.5	17.1	28.2	7.6	21.8	4.1	21.3	2.6	6.1	1.4	11.0	1.5	3.4
2016	3.3	13.4	16.1	28.2	7.8	22.1	3.8	21.6	2.4	6.4	1.1	10.9	1.5	3.5
2017	3.0	13.2	16.0	27.4	7.9	22.1	3.6	21.6	2.3	7.0	1.1	11.3	1.8	3.5
2018	2.8	12.7	15.7	27.4	8.0	22.6	3.4	21.6	2.0	6.7	1.2	11.0	1.7	3.5
2019	2.8	12.8	15.2	27.9	8.9	23.2	3.4	22.0	2.3	6.7	1.3	10.7	1.9	3.1

Note: All rates are per 100,000 years and have been age standardised to CPRD population as of 1/1/2019
