# Supplementary material for: Prevalence and incidence of neuromuscular conditions in the UK between 2000 and 2019: A retrospective study using primary care data
Source: PLoS One. 2021 Dec 31;16(12):e0261983. doi: 10.1371/journal.pone.0261983 (PMC8719665; doi:10.1371/journal.pone.0261983)
Supplement: S20 Table — (PDF) [file pone.0261983.s020.pdf]

Table S20 – Age standardised prevalence rates 2000-19 for selected conditions, ages 45- only

Year	Inflammatory myopathies		Muscular dystrophies		Charcot-Marie Tooth disease		Guillain-Barré syndrome				Myasthenia gravis		Motor neurone disease	
							Lifetime		Code in last 5 years					
	45-64y	65-y	45-64y	65-y	45-64y	65-y	45-64y	65-y	45-64y	65-y	45-64y	65-y	45-64y	65-y
2000	23.3	33.4	31.1	18.8	17.0	16.0	34.5	46.9	9.9	13.4	24.6	45.8	14.7	34.1
2001	22.8	34.7	31.9	20.9	17.2	17.7	35.9	50.1	10.4	14.6	25.0	48.0	15.4	33.5
2002	24.8	35.6	32.4	22.2	18.1	19.1	36.7	53.6	10.1	15.7	26.2	52.0	15.0	34.2
2003	26.2	37.3	33.9	23.6	18.4	20.2	38.2	56.7	10.0	16.9	26.5	55.7	15.1	35.4
2004	26.9	38.6	35.1	25.2	20.1	21.9	41.3	58.2	11.2	16.8	28.1	61.0	15.1	35.5
2005	28.7	41.3	37.0	26.9	21.5	25.3	42.8	60.7	11.7	16.9	29.2	63.8	13.9	38.9
2006	29.0	42.5	37.9	28.5	23.4	28.7	43.9	63.8	11.9	16.8	29.9	67.5	14.2	38.1
2007	29.3	44.4	38.2	30.2	24.9	32.2	44.7	66.3	12.1	17.0	30.1	71.0	13.6	37.7
2008	29.9	45.8	38.7	30.5	26.6	33.5	46.2	68.2	12.1	17.2	30.3	71.7	13.7	35.8
2009	30.4	47.7	38.8	31.4	28.0	35.8	47.9	70.6	12.2	17.6	31.2	76.1	14.4	36.3
2010	30.8	49.0	38.3	32.8	29.5	37.8	49.4	72.8	12.2	18.5	30.9	80.5	15.0	37.5
2011	30.9	51.1	38.9	32.4	31.0	39.0	50.5	73.9	12.7	18.0	31.4	82.0	14.5	38.3
2012	30.7	51.2	38.5	34.0	31.5	40.5	50.6	77.3	12.6	19.2	32.1	84.8	15.1	38.5
2013	30.8	52.4	38.3	34.8	32.6	40.8	51.2	80.2	12.8	20.2	32.7	87.3	14.6	39.6
2014	32.1	53.4	38.9	34.6	33.5	42.6	51.8	84.1	12.6	21.0	32.7	90.8	14.1	39.3
2015	31.9	54.2	38.1	35.1	34.7	43.7	52.6	84.6	12.3	20.4	33.3	94.2	13.8	37.6
2016	32.9	54.3	37.7	34.9	35.4	44.9	52.7	85.9	12.5	20.7	34.0	97.1	14.7	38.5
2017	33.3	55.9	36.6	34.9	35.7	47.2	53.3	87.4	13.0	20.3	34.7	101.1	14.8	38.3
2018	33.8	57.5	36.2	35.5	36.7	48.0	52.8	90.6	13.0	22.3	35.6	102.1	14.1	37.4
2019	32.8	58.5	36.2	35.8	37.6	49.2	53.0	91.6	12.9	22.0	36.8	105.3	15.1	37.9

Note: All rates are per 100,000 years and have been age standardised to CPRD population as of 1/1/2019
