# Supplementary material for: Prevalence and incidence of neuromuscular conditions in the UK between 2000 and 2019: A retrospective study using primary care data
Source: PLoS One. 2021 Dec 31;16(12):e0261983. doi: 10.1371/journal.pone.0261983 (PMC8719665; doi:10.1371/journal.pone.0261983)
Supplement: S22 Table — (PDF) [file pone.0261983.s022.pdf]

Table S22 – Age standardised incidence rates 2000-19 for selected conditions, ages 45- only

Year	Inflammatory myopathies		Muscular dystrophies		Charcot-Marie Tooth disease		Guillain-Barré syndrome		Myasthenia gravis		Motor neurone disease	
	**45-64y**	**65-y**	**45-64y**	**65-y**	**45-64y**	**65-y**	**45-64y**	**65-y**	**45-64y**	**65-y**	**45-64y**	**65-y**
2000	2.03	2.55	2.03	2.16	0.89	1.12	2.73	3.26	1.87	3.83	4.33	10.06
2001	2.61	3.24	2.02	1.68	1.54	1.38	1.95	3.17	3.01	4.83	3.79	9.44
2002	3.17	2.23	1.91	1.82	0.80	1.64	2.04	3.39	1.73	6.03	4.05	11.29
2003	2.09	2.15	2.42	2.12	1.99	1.69	2.84	2.91	2.24	6.48	3.63	10.25
2004	2.44	4.42	2.27	1.92	2.07	2.57	2.06	2.09	1.95	4.80	3.39	11.46
2005	2.10	2.85	1.88	1.64	1.94	2.74	1.68	2.63	2.19	6.13	4.24	11.69
2006	2.03	3.14	1.79	1.81	1.75	2.91	2.00	2.80	2.35	6.25	3.17	11.58
2007	2.23	3.23	1.25	1.49	2.19	2.83	2.28	2.51	1.75	4.77	3.99	9.93
2008	2.02	2.77	1.59	1.71	1.76	2.86	2.50	3.21	2.68	7.03	3.89	10.19
2009	2.06	2.82	1.23	1.83	1.56	1.98	2.23	3.77	2.25	6.46	3.95	12.01
2010	1.96	2.84	1.36	1.55	1.64	1.87	2.15	2.61	2.15	5.70	4.06	13.02
2011	1.57	1.79	1.79	1.51	1.14	2.32	2.50	3.13	2.08	6.76	3.98	11.41
2012	1.54	2.99	1.60	1.76	1.37	1.60	1.91	3.26	2.27	6.82	3.62	13.07
2013	1.74	2.26	1.06	1.20	1.61	1.76	2.27	3.42	1.96	6.90	3.89	13.68
2014	1.50	1.98	1.17	1.01	1.51	1.37	1.84	2.76	2.71	7.98	3.15	12.72
2015	2.17	2.25	1.03	1.32	1.27	2.37	2.51	2.93	2.30	8.23	4.73	13.55
2016	2.03	2.59	0.77	1.10	1.42	2.36	2.21	2.99	2.82	7.55	3.82	12.68
2017	1.99	3.20	1.34	0.98	2.05	2.15	2.44	3.31	2.77	7.09	3.43	11.98
2018	1.56	1.97	1.22	1.00	1.61	1.66	2.51	3.03	2.83	9.36	3.73	13.10
2019	1.83	2.92	1.01	1.04	1.22	2.55	1.36	3.02	2.30	7.95	3.66	11.19

Note: All rates are per 100,000 years and have been age standardised to CPRD population as of 1/1/2019
