# Supplementary material for: Prevalence and incidence of neuromuscular conditions in the UK between 2000 and 2019: A retrospective study using primary care data
Source: PLoS One. 2021 Dec 31;16(12):e0261983. doi: 10.1371/journal.pone.0261983 (PMC8719665; doi:10.1371/journal.pone.0261983)
Supplement: S23 Table — (PDF) [file pone.0261983.s023.pdf]

Table S23: Population standardised prevalence (2019) and incidence (2015-9) of recorded neuromuscular disease in the UK with estimated number of people

Classification	Prevalence (2019)		Incidence (2015-9)	
	Rate per 100,000 persons (95% CI)	Estimated no. of people	Rate per 100,000 person yrs. (95% CI)	Estimated no. of people
**Motor Neuron Disorders**				
- Motor neurone disease (MND)	12.4 (11.8-13.0)	8,273	3.49 (3.34-3.64)	2,331
- Post polio syndrome	3.7 (3.4-4.0)	2,461	0.13 (0.10-0.16)	85
- Spinal muscular atrophy	4.9 (4.6-5.3)	3,303	0.20 (0.17-0.24)	135
**Muscle Disease**				
Acquired myopathies				
- Endocrine myopathy	0.5 (0.3-0.6)	306	0.01 (0.00-0.02)	9
- Infectious myopathy	3.4 (3.1-3.7)	2,279	0.21 (0.17-0.24)	139
- Inflammatory myopathies (IIM)	24.7 (23.8-25.5)	16,483	1.30 (1.21-1.39)	870
- Toxic or drug-induced myopathy	2.5 (2.2-2.8)	1,685	0.21 (0.18-0.25)	142
Hereditary myopathies				
- Congenital myopathy	3.1 (2.8-3.4)	2,058	0.22 (0.18-0.25)	144
- Metabolic myopathies	5.6 (5.2-6.0)	3,756	0.25 (0.21-0.29)	166
- Muscular dystrophy (MD)	29.1 (28.2-30.1)	19,469	1.17 (1.08-1.25)	780
Muscular dystrophy type				
- Duchenne MD	3.1 (2.8-3.4)	2,085	0.15 (0.11-0.18)	97
- Becker MD	2.0 (1.8-0.0)	1,358	0.12 (0.09-0.14)	78
- Limb-girdle MD*	1.6 (1.2-1.6)	1,093	0.07 (0.05-0.09)	47
- Facioscapulohumeral MD	3.0 (2.7-3.3)	2,035	0.15 (0.12-0.18)	99
- Myotonic dystrophy type 1†	9.2 (8.6-9.7)	6,117	0.43 (0.38-0.49)	290
Other				
- Mitochondrial disease	4.4 (4.1-4.8)	2,953	0.29 (0.25-0.34)	197
- Myotonic disorders (non-dystrophic)	2.6 (2.3-2.9)	1,737	0.12 (0.09-0.15)	80
- Myotonic disorders (unspecified)	1.2 (1.0-1.4)	820	0.04 (0.02-0.05)	25
- Periodic paralysis	3.2 (2.9-3.5)	2,114	0.21 (0.18-0.25)	143
**Neuropathies**				
Hereditary neuropathy				
- Charcot-Marie Tooth (CMT)	29.0 (28.1-29.9)	19,376	1.45 (1.35-1.54)	967
- Other hereditary neuropathy	0.6 (0.5-0.8)	431	0.02 (0.01-0.03)	14
Inflammatory & autoimmune neuropathies				
- Guillain-Barré syndrome (GBS)	39.3 (38.2-40.4)	26,253	1.70 (1.60-1.80)	1,136
**-** GBS recorded in last 5 years only	10.3 (9.8-10.9)	6,891		
- Other inflammatory & autoimmune neuropathies	12.8 (12.2-13.4)	8,554	0.69 (0.63-0.76)	463

Classification	Prevalence (2019)		Incidence (2015-9)	
	**Rate per 100,000 persons (95% CI)**	**Estimated no. of people**	**Rate per 100,000 person yrs. (95% CI)**	**Estimated no. of people**
**Neuromuscular Junction Disorders**				
- Eaton-Lambert syndrome	0.3 (0.2-0.4)	231	0.03 (0.02-0.05)	23
- Myasthenia gravis (MG)	33.1 (32.1-34.1)	22,119	2.49 (2.37-2.62)	1,667
- Other NMJ disorder‡	3.2 (2.9-3.5)	2,126	0.13 (0.10-0.16)	86
Muscular or neuromuscular disease unspecified*	7.3 (6.8-7.7)	4,859	0.52 (0.46-0.57)	345
**All Neuromuscular Disease**				
**-** Any	220.3 (217.7-222.9)	147,163	14.22 (13.92-14.53)	9,501
- Any, only with GBS in last 5 years	191.9 (189.5-194.3)	128,189		

Note: Rates have been age-sex standardised to ONS 2019 mid-year population estimates for the whole of the UK. Patients can belong to multiple categories except for "unspecified", where they are only classified if no other appropriate classification was available.
