# Supplementary material for: Prevalence and incidence of neuromuscular conditions in the UK between 2000 and 2019: A retrospective study using primary care data
Source: PLoS One. 2021 Dec 31;16(12):e0261983. doi: 10.1371/journal.pone.0261983 (PMC8719665; doi:10.1371/journal.pone.0261983)
Supplement: S24 Table — (PDF) [file pone.0261983.s024.pdf]

Table S24 – Estimated numbers of people in UK with recorded neuromuscular disease compared with published estimates for Parkinson's and multiple sclerosis using other CPRD data

Classification	Year	Standardised Rates* per 100,000			Estimated no. of people		
		All	Females	Males	All	Females	Males
**Prevalence**							
All Neuromuscular Disease	2019	220.3	206.2	234.8	147,163	69,732	77,431
	2019, only with GBS codes in last 5 years	191.9	179.6	204.5	128,198	60,747	67,451
Parkinson's	2015	210.1	175.1	246.2	136,816	57,854	78,963
Multiple Sclerosis	2010	203.4	289.2	114.9	126,669	91,444	35,225
**Incidence**							
All Neuromuscular Disease	2015-19	14.2	12.5	16.0	9,501	4,191	4,891
Parkinson's	2011-15	26.6	20.4	33.0	17,314	6,744	10,569
Multiple Sclerosis	1990-2010	9.6	13.4	5.7	6,003	4,250	1,754

* - Rates have all been age-sex standardised to national UK population for the individual study periods. Parkinson's figure taken from the Parkinson's UK 2017 report [1]. Multiple sclerosis taken from Mackenzie et al [2].

1. Parkinson's UK. The prevalence and incidence of Parkinson's in the UK. 2017: https://www.parkinsons.org.uk/sites/default/files/2018-01/Prevalence%20%20Incidence%20Report%20Latest_Public_2.pdf. Accessed 27-08-2021.
2. Mackenzie IS, Morant SV, Bloomfield GA, MacDonald TM, O'Riordan J. Incidence and prevalence of multiple sclerosis in the UK 1990-2010: a descriptive study in the General Practice Research Database. *J Neurol Neurosurg Psychiatry* 2014;85(1):76-84.
